# Immediate effects of hybrid assistive limb gait training on lower limb function in a chronic myelopathy patient with postoperative late neurological deterioration

**DOI:** 10.1186/s13104-022-05979-4

**Published:** 2022-03-04

**Authors:** Shigeki Kubota, Hideki Kadone, Yukiyo Shimizu, Masao Koda, Hiroshi Takahashi, Kousei Miura, Fumihiko Eto, Takeo Furuya, Yoshiyuki Sankai, Masashi Yamazaki

**Affiliations:** 1 Department of Orthopaedic Surgery, Faculty of Medicine, University of Tsukuba, 1-1-1 Tennodai, Tsukuba, Ibaraki 305-8575 Japan; 2 Center for Innovative Medicine and Engineering, University of Tsukuba, 1-1-1 Tennodai, Tsukuba, Ibaraki 305-8575 Japan; 3 Department of Rehabilitation Medicine, Faculty of Medicine, University of Tsukuba, Ibaraki, 305-8575 Japan; 4 Department of Orthopaedic Surgery, Chiba University Graduate School of Medicine, Chiba, 260-8677 Japan; 5 Faculty of Engineering, Information and Systems, University of Tsukuba, 1-1-1 Tennodai, Tsukuba, Ibaraki 305-8573 Japan

**Keywords:** Wearable electronic devices, Gait, Exercise therapy, Spinal cord diseases

## Abstract

**Objective:**

The Hybrid Assistive Limb (HAL) has recently been used to treat movement disorders. Although studies have shown its effectiveness for chronic myelopathy, the immediate effects of HAL gait training on lower limb function have not been clarified. We conducted HAL gait training and examined its immediate effects on a 69-year-old man with re-deterioration of myelopathy in the chronic phase after surgery for compression myelopathy. The HAL intervention was performed every 4 weeks for 10 total sessions. Immediately before and after each session, we analyzed the patient’s walking ability using the 10-m walk test. In the 4th HAL session, the gastrocnemius muscle activity was measured bilaterally using a synchronized motion capture-electromyogram system.

**Results:**

The training effects became steady after the 2nd session. In sessions 2–10, the step length increased from 0.56 to 0.63 m (mean: 0.031 m) immediately after HAL training. The motion capture-electromyogram analyses showed that considerable amounts of gastrocnemius muscle activity were detected during the stance and swing phases before HAL training. During and immediately after HAL training, gastrocnemius activity during the swing phase was diminished. HAL gait training has an immediate effect for inducing a normal gait pattern with less spasticity in those with chronic myelopathy.

**Supplementary Information:**

The online version contains supplementary material available at 10.1186/s13104-022-05979-4.

## Introduction

The Hybrid Assistive Limb (HAL®), a wearable exoskeleton robot, can assist in the voluntary control of knee and hip joint motions [1, 2]. The HAL provides physical support according to the wearer's voluntary intention by detecting his/her electrical signals, including muscle activity. Patients with spinal cord disorders cannot move their bodies because intention signals from the brain are not appropriately transmitted from the injured spinal cord to the more distant body parts. The HAL captures faint myoelectrical signals in the periphery, and the HAL power unit generates sufficient assist torque by amplifying the patient’s diminished joint torque. Thus, HAL can provide motion support for patients with spinal cord disorders. Previous studies have shown the effectiveness of HAL gait training for several spinal conditions, including spinal cord injury [3], acute and chronic stages after surgery for compression myelopathy [4–6], arteriovenous malformation [7], and spinal cord infarction [8]. However, there is no report on the immediate effect of HAL gait training. We conducted HAL gait training for a patient with re-deterioration of myelopathy in the chronic phase after surgery and examined the immediate effects of HAL gait training.

## Main text

### Patient

**Fig. 1:**
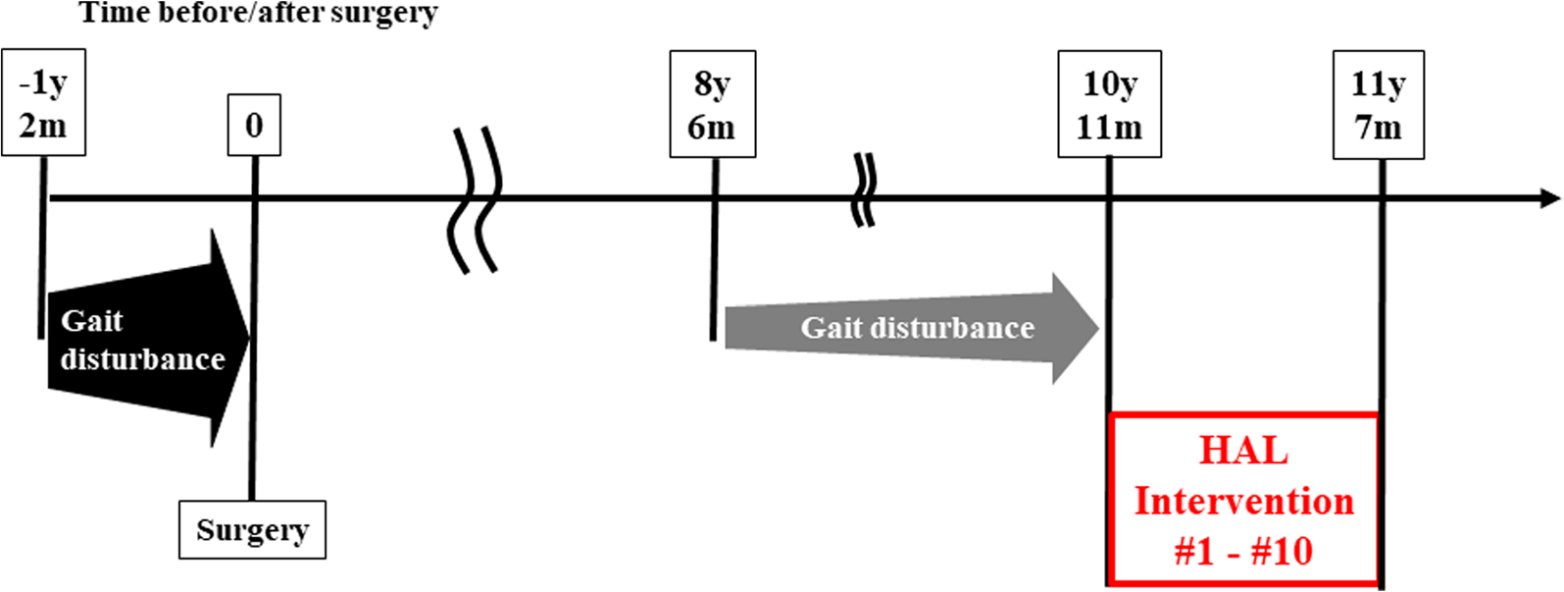
The patient’s symptom progression and the point in time at which Hybrid Assistive Limb training was performed

A 69-year-old man presented with late deterioration of myelopathy following spine surgery and underwent HAL gait training to improve his ability to walk. The progression of the gait dysfunction is summarized in Fig. 1.

### Preoperative clinical data

The patient initially presented with clumsiness of his right hand and rapidly progressing gait disturbance. Imaging findings revealed compression myelopathy due to cervical ossification of the posterior longitudinal ligament (OPLL) and thoracic ossification of the ligamentum flavum (OLF) (Additional file 1).

### Surgery and postoperative course

Posterior decompression surgery was performed for the compressed spinal cord. We performed laminoplasty at C4–C6, laminotomy of the inferior portion of C3 and cranial portion of C7, and laminectomy at T3–T8. Postoperative imaging findings showed that adequate decompression of the spinal cord was achieved at the thoracic spine, but slight compression of the cervical spinal cord remained at the C3–C4 level (Additional file 2). Postoperatively, the myelopathy was relieved. However, 8 years and 6 months after surgery, he experienced deterioration of his gait, although no worsening of upper extremity function was noted (Fig. 1). He experienced an increase in spasticity in the right leg. Analyses of magnetic resonance (MR) images showed atrophic changes in the spinal cord at the C4–C5 level (Additional file 3).

### HAL intervention

Ten years and 11 months after surgery, the patient underwent HAL gait training. Approximately every 4 weeks, he underwent HAL gait training and completed 10 sessions (Fig. 1). The HAL intervention was provided by a medical doctor, who was present in case of emergency, as well as a therapist, two assistants, and an engineer, as reported previously [6, 9]. Each HAL session lasted 60 min, including the time for attaching/detaching the device, rest, and walking along a 25-m-long circuit several times. The net gait training time was approximately 20 min. The patient did not use any assistive walking devices, such as a t-cane, lateral crutch, or walker in his daily life and during the HAL training. In the HAL session, the operator adjusted the degree of physical support provided to the patient. Table 1 shows the assist settings for the patients’ hip and knee joints in each HAL session. Higher values of the HAL assist setting indicate greater physical support provided to the patient.

**Table 1:** Assist setting for the patient's hip and knee joints at each HAL session

HAL session	Hip				Knee			
	Right		Left		Right		Left	
	Flex	Ext	Flex	Ext	Flex	Ext	Flex	Ext
1	8.4	12	10	10	12	12	7	10
2	6	10	5.4	9	10	10	5.4	9
3	6	10	5.4	9	7	10	2.7	9
4	4	10	5.4	9	10	10	6.3	9
5	3	10	2.7	9	10	10	6.3	9
6	5	10	4.5	9	10	10	6.3	9
7	5	10	4.5	9	10	10	6.3	9
8	5	10	4.5	9	10	10	6.3	9
9	5	10	4.5	9	10	10	6.3	9
10	5	10	4.5	9	10	10	6.3	9

The assist setting is expressed as the value on a scale of 0–20.

Flex, flexion; Ext, extension

### Functional evaluation

A 10-m walk test and 2-min walk test were conducted before (baseline) and after (after training) the HAL intervention. The initial testing (baseline) was performed 24 days before the 1st HAL session. The final test (after training) was performed 5 days after the 10th HAL session. During the 10-m walk test, the patient was instructed to walk without wearing the HAL on a flat surface at a self-selected comfortable pace. Walking speed (m/min), step length (m), and cadence (steps/min) were calculated as previously described [6, 9]. During the 2-min walk test, the patient was asked to walk for 2 min at a chosen maximal pace, and the total distance walked was recorded. The 10-m walk test was also performed in each HAL session just before and after detaching the HAL to evaluate the immediate effects of the HAL training on lower limb functions.

Gait characteristics were measured using a VICON motion capture system (Vicon MX System with 16 T20s cameras; Oxford Metrics Ltd., Oxford, United Kingdom) three times during each session as follows: unassisted gait just before training with the robot (pre-HAL), gait while ambulating with the wearable robot during training (HAL), and unassisted gait after removal of the robot after training (post-HAL). During these tests, the patient was instructed to walk on a flat surface at a self-selected, comfortable pace. Auto-reflexive markers were attached to the feet following the VICON plug-in gait marker placement on the foot. The toe lift was computed according to the relative height displacement measured by the maximum height minus the minimum height of a toe marker for each step and then averaged among the extracted steps. The step length and toe lift were computed separately for the right and left sides, focusing on lateral symmetry.

The activity of the gastrocnemius muscle was recorded bilaterally using a Trigno Lab wireless electromyography (EMG) system (Delsys, United States). Because of the spasticity of the ankle plantar flexors, the patient had foot drop during the swing phase. Therefore, the phase-dependent muscle activation of the plantar flexors was evaluated using the ratio of the integrated activity of the gastrocnemius during the swing phase to the activity over the entire step cycle (combined swing and stance phases).

## Results

### Change in walking ability at baseline and after the HAL intervention

Table 2 shows the data of the 10-m and 2-min walk tests at baseline and after the HAL intervention.

**Table 2:** Walking ability at baseline and after the HAL intervention

	At baseline	After the HAL intervention
10-m walk test		
Speed (m/sec)	1.06	1.31
Step length (m)	0.56	0.65
Cadence (steps/min)	114.6	121.9
2-min walk test		
Total walking distance (m)	142.5	156.6

At baseline: data were obtained 24 days before the 1st HAL session

After the HAL intervention: data were obtained 5 days after the 10th HAL session

After the HAL intervention, the maximum hip angle flexion during the swing phase (mean ± SD) increased from 23.3 ± 0.4° to 32.5 ± 1.2° on the right side and from 27.6 ± 0.8° to 39.7 ± 2.2° on the left side (Additional file 4A, B). Similarly, maximum flexion decreased at the knee from 70.6 ± 3.0° to 63.7 ± 3.3° on the right side and from 65.8 ± 1.7° to 65.8 ± 2.2° on the left side (Additional file 5A, B).

### Change in walking ability immediately before and after HAL gait training in each HAL session

Additional file 6 shows the data of the 10-m walk test immediately before and after the HAL gait training in each HAL session. The patient’s gait speed after the HAL training was increased in sessions 2, 3, 5, 7, 8, and 10, did not change in sessions 4 and 6, and decreased in sessions 1 and 9 (Additional file 6A). The step length did not change immediately before and after HAL training in session 1. In sessions 2–10, the step length was increased from 0.56 m to 0.63 m (mean: 0.031 m) immediately after HAL training (Additional file 6B). The cadence slightly increased in session 7 and decreased in sessions 1–6 and 8–10 (Additional file 6C).

### Results of kinematic motion analysis for the 4th HAL session

The changes in the lower limb kinematics are shown in Additional file 7, 8, 9. Although step length increased from 0.51 ± 0.03 m to 0.57 ± 0.02 m on the right side, the left side was unchanged (from 0.59 ± 0.02 m to 0.59 ± 0.02 m) (Additional file 7). Additional file 8 shows data for the toe lift of the right and left legs. In the right leg, it was 7.6 ± 0.8 cm before, 12.7 ± 4.6 cm during, and 9.5 ± 0.8 cm after the HAL training. In the left leg, it was 10.9 ± 0.9 cm before, 11.0 ± 2.4 cm during, and 12.4 ± 1.1 cm after the HAL training (Additional file 8).

Surface EMG profiles of the gastrocnemius muscle during the swing and stance phases are shown in Additional file 9. The activity of the gastrocnemius muscle was evident in the right leg during the swing phase (Additional file 9A), but the activity in the left leg during the swing phase was diminished during and after HAL training. Additional file 9B shows the ratio of the integrated activity of the gastrocnemius muscle during the swing phase to the activity over the entire step cycle (combined swing and stance phases). In the right leg, it was 1.90 ± 0.77 mV before, 0.21 ± 0.07 mV during, and 0.37 ± 0.13 mV after the HAL training. In the left leg, it was 1.79 ± 0.45 mV before, 0.76 ± 0.29 mV during, and 0.60 ± 0.15 mV after the HAL training.

## Discussion

### Pathophysiology of late neurological deterioration

We have occasionally encountered late neurological deterioration after surgery for compression myelopathy despite repeated adequate decompression of the spinal cord. We previously reported two cases of late neurological deterioration after decompression surgery for myelopathy due to cervical OPLL and thoracic OLF [6, 9]. The myelopathy symptoms were relieved after surgery but began to deteriorate without any apparent cause at 2 years and 15 years after surgery, respectively. The T2-weighted MR images (T2WI) of the decompressed spinal cords were characteristic. The spinal cords were markedly decompressed, but high-intensity changes appeared inside the spinal cord, which was atrophic.

We previously reported an autopsy case [10] in which laminoplasty for cervical spondylotic myelopathy was performed 9 years before the autopsy. T2WI showed a high-intensity area having a “snake-eyed” appearance at the decompressed spinal cord. Although such abnormal findings were predominantly observed in the gray matter by the MR image analyses, subsequent histopathological analyses demonstrated that atrophic changes in the spinal cord were evident in both the gray and white matter. Considering this and previous studies that analyzed the histopathology of damaged spinal cords in patients with compression myelopathy [11, 12], we suggest that, in the damaged spinal cord showing such “snake-eyed” appearance by MR image analyses, demyelination of the funiculus may progress in the white matter, and the spinal cord is in a condition of atrophy.

In the present case, the patient had myelopathy symptoms in both the upper and lower extremities preoperatively, and his symptoms were especially severe in his right leg. After surgery, the symptoms in both the upper and lower extremities were relieved. Although atrophic changes in the spinal cord existed at the C4–C5 level based on MR image analyses, late neurological deterioration developed only in his right leg. This suggests that, in the present case, atrophic changes in the white matter, such as the demyelination of the funiculus, existed at the C4–C5 level and may have participated in the development of the late neurological deterioration.

To date, there is no effective treatment for such late neurological deterioration caused by spinal cord atrophy. We previously performed gait training with HAL for late neurological deterioration after surgery for compression myelopathy, and obtained considerable recovery of gait function [6, 9]. Thus, in the present case, we chose HAL gait training for the recovery of gait function.

### Effects of HAL gait training on lower limb function

In the present case, the patient’s walking ability was enhanced after 8 months of HAL intervention. The effect of HAL on improving gait in the present case was consistent with that of previous cases [6, 9], suggesting that repeated and long-term HAL gait training may cause the recovery of lower limb function in patients with chronic myelopathy. We further analyzed the HAL effect using motion capture and wireless EMG systems. Because the present patient showed severe paralysis in his right leg, we set a stronger assist level on the right side. Thus, the effect of HAL was detected predominantly in the right leg. The height of the toe lift was greater during the HAL gait training than before the HAL training (Additional file 8). Although the HAL does not have the function of assisting ankle motion, it can increase hip flexion and knee extension during training. This may be one of the reasons why toe lift increased during the HAL gait training. In addition, during the training, contraction of the gastrocnemius muscle decreased during the swing phase (Additional file 9), possibly also contributing to increased toe lift.

Notably, the increased toe lift and decreased contraction of the gastrocnemius muscle during the swing phase were maintained immediately after the HAL training. We suggest that exercise memory during the HAL training was retained in the patient’s brain, and he was able to reproduce the improved gait pattern after the HAL training even without HAL. It is possible that his brain relearned the normal gait pattern by the voluntary walking exercise with HAL.

## Limitations

This study is a single case report and could not compare the efficacy of the HAL training with that of conventional rehabilitation.

## Supplementary Information

**Additional file 1**: Reconstruction images from computed tomography (CT) myelography of the cervical and thoracic spine before surgery. Midsagittal reconstruction CT myelogram of the cervical spine (A) and axial CT image at the C5 level (B) reveal segmental ossification of the longitudinal ligament (OPLL) at the C4–C6 vertebrae. The asterisks in (A) and (B) indicate the C5 OPLL. The spinal cord was compressed from the anterior and posterior directions at the C3–C4 and C4–C5 levels (red arrows). Midsagittal reconstruction CT myelogram of the thoracic spine (C) and axial CT image at the T4–T5 level (D) reveal multilevel ossification of the ligamentum flavum (OLF) at the T3–T8 vertebrae. The asterisks in (C) and (D) indicate the T4–T5 OLF. The spinal cord was compressed posteriorly at the T4–T5 level (blue arrow).

**Additional file 2:** Magnetic resonance (MR) images of the cervical and thoracic spine 14 months after surgery. T1-weighted (A) and T2-weighted (B) midsagittal MR images show sufficient decompression of the spinal cord at the thoracic spine. Magnification of the T2-weighted image at the cervical spine area (C) shows slight posterior compression of the spinal cord at the C3–C4 level (red arrow).

**Additional file 3:** MR images of the cervical spine 10 years and 6 months after surgery. T1-weighted (A) and T2-weighted (B) midsagittal MR images show slight posterior compression of the spinal cord at the C3–C4 level (red arrows). In the T2-weighted MR image (B), a high-intensity area was present inside the spinal cord at the C4–C5 level (red arrowhead), although the cord was thoroughly decompressed at this level. A T2-weighted MR axial image at the C4–C5 level (C) shows that the high-intensity area was predominant in the gray matter with a "snake-eyed" appearance (blue arrowheads).

**Additional file 4:** Data of the kinematic motion analysis of the hip joint for HAL session 4. (A) Temporal profile of the angular position of the hip joint over the gait cycle (A) and range of motion of the hip over the gait cycle (B), measured without, immediately before, and after the HAL training. Error bars indicate standard error of the mean. Flex, flexion; Ext, extension; Pre, Pre-HAL training; Post, Post-HAL training; ROM, range of motion.

**Additional file 5:** Data of the kinematic motion analysis of the knee joint for HAL session 4. (A) Temporal profile of the angular position of the knee joint over the gait cycle (A) and range of motion of the knee over the gait cycle (B), measured without, immediately before, and after the HAL training. Error bars indicate standard error of the mean. Flex, flexion; Ext, extension; Pre, Pre-HAL training; Post, Post-HAL training; ROM, range of motion.

**Additional file 6:** The 10-m walk test data immediately before and after HAL gait training, sessions 1–10. Gait speed (A), step length (B), and cadence (C).

**Additional file 7:** Data of the kinematic motion analysis of step length for HAL session 4. Step length of the right and left legs immediately before and after HAL training. Pre, Pre-HAL training; Post, Post-HAL training.

**Additional file 8:** Data of the kinematic motion analysis of toe clearance for HAL session 4. Toe lift of the right and left legs before, during, and after HAL gait training. The toe lift indicates toe clearance. Pre, Pre-HAL training; Post, Post-HAL training.

**Additional file 9:** Kinematic motion analysis using the VICON motion capture system and surface electromyography (HAL session 4). (A) Surface electromyography of the gastrocnemius muscles of the right and left legs during the stance and swing phases. (B) Gastrocnemius stance ratio of the right and left legs before, during, and after the HAL gait training. The gastrocnemius stance ratio indicates the muscle activation ratio of the gastrocnemius (swing phase to the total step cycle). Pre, Pre-HAL training; Post, Post-HAL training.

## Data Availability

All data generated or analyzed during this study are included in this published article [and its additional files].

## Abbreviations

EMG: Electromyography
HAL: Hybrid assistive limb
MR: Magnetic resonance
OLF: Ossification of the ligamentum flavum
OPLL: Ossification of the posterior longitudinal ligament
T2WI: T2-weighted MR images
