# Supplementary figures and images for: Immediate effects of hybrid assistive limb gait training on lower limb function in a chronic myelopathy patient with postoperative late neurological deterioration
Source: BMC Res Notes. 2022 Mar 4;15:89. doi: 10.1186/s13104-022-05979-4 (PMC8896224; doi:10.1186/s13104-022-05979-4)

## Slide 1
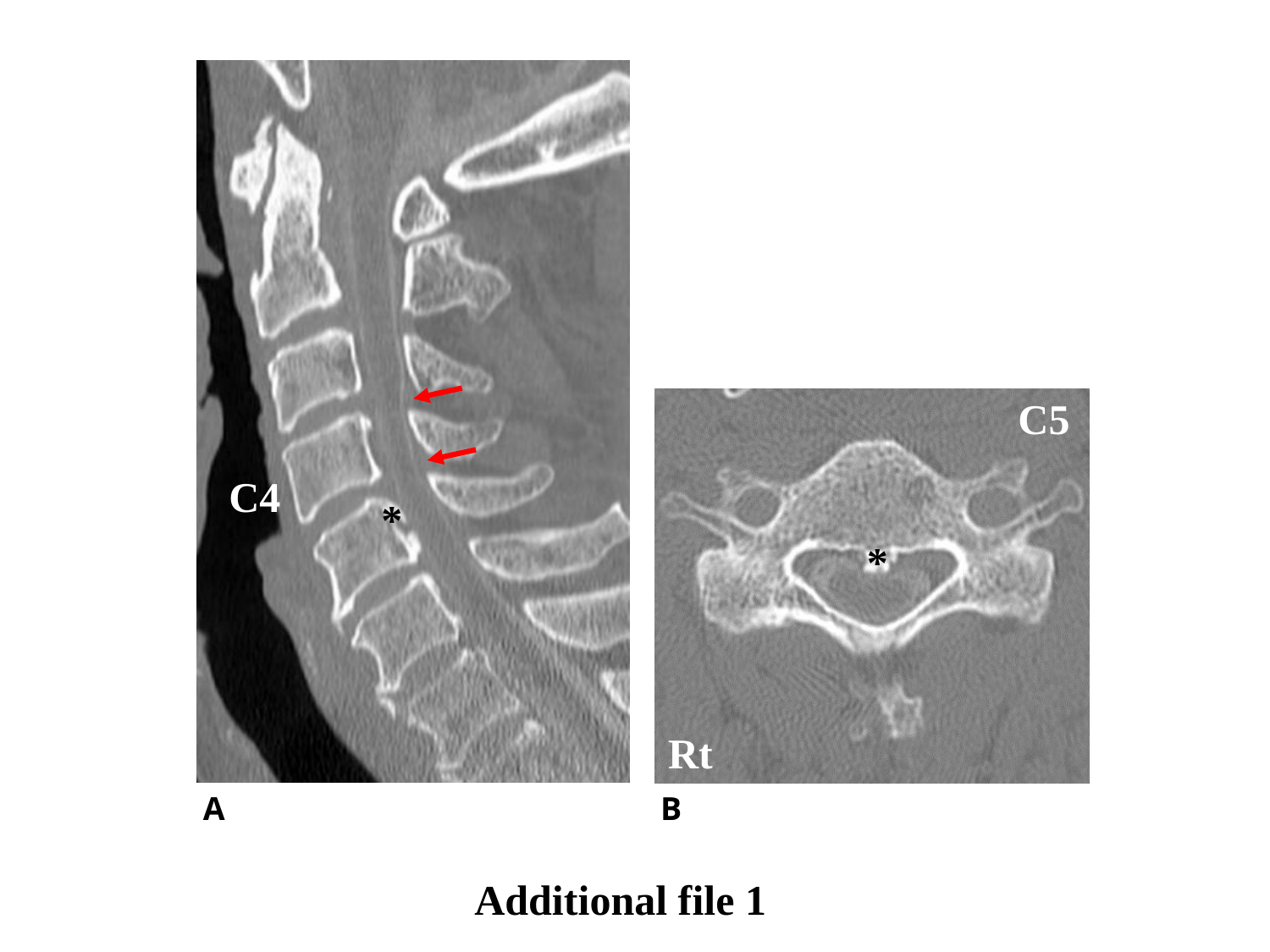

C5
C4
*
*
Rt
A
B
Additional file 1

## Slide 2
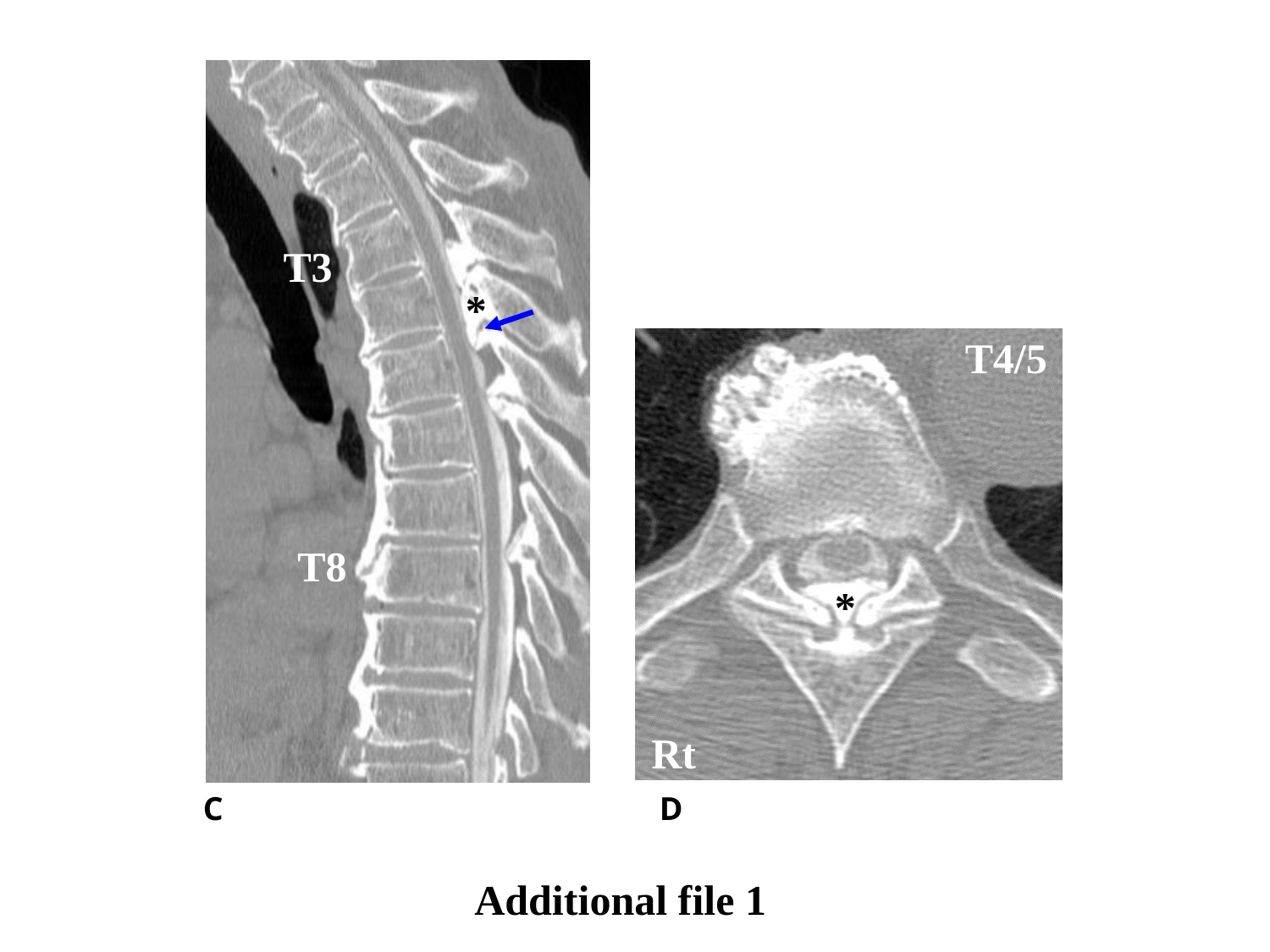

T3
*
T4/5
T8
*
Rt
C
D
Additional file 1

Supplement: Supplementary file 1 — Additional file 1: Reconstruction images from computed tomography (CT) myelography of the cervical and thoracic spine before surgery. Midsagittal reconstruction CT myelogram of the cervical spine (A) and axial CT image at the C5 level (B) reveal segmental ossification of the longitudinal ligament (OPLL) at the C4–C6 vertebrae. The asterisks in (A) and (B) indicate the C5 OPLL. The spinal cord was compressed from the anterior and posterior directions at the C3–C4 and C4–C5 levels (red arrows). Midsagittal reconstruction CT myelogram of the thoracic spine (C) and axial CT image at the T4–T5 level (D) reveal multilevel ossification of the ligamentum flavum (OLF) at the T3–T8 vertebrae. The asterisks in (C) and (D) indicate the T4–T5 OLF. The spinal cord was compressed posteriorly at the T4–T5 level (blue arrow). [file 13104_2022_5979_MOESM1_ESM.pptx]

## Slide 1
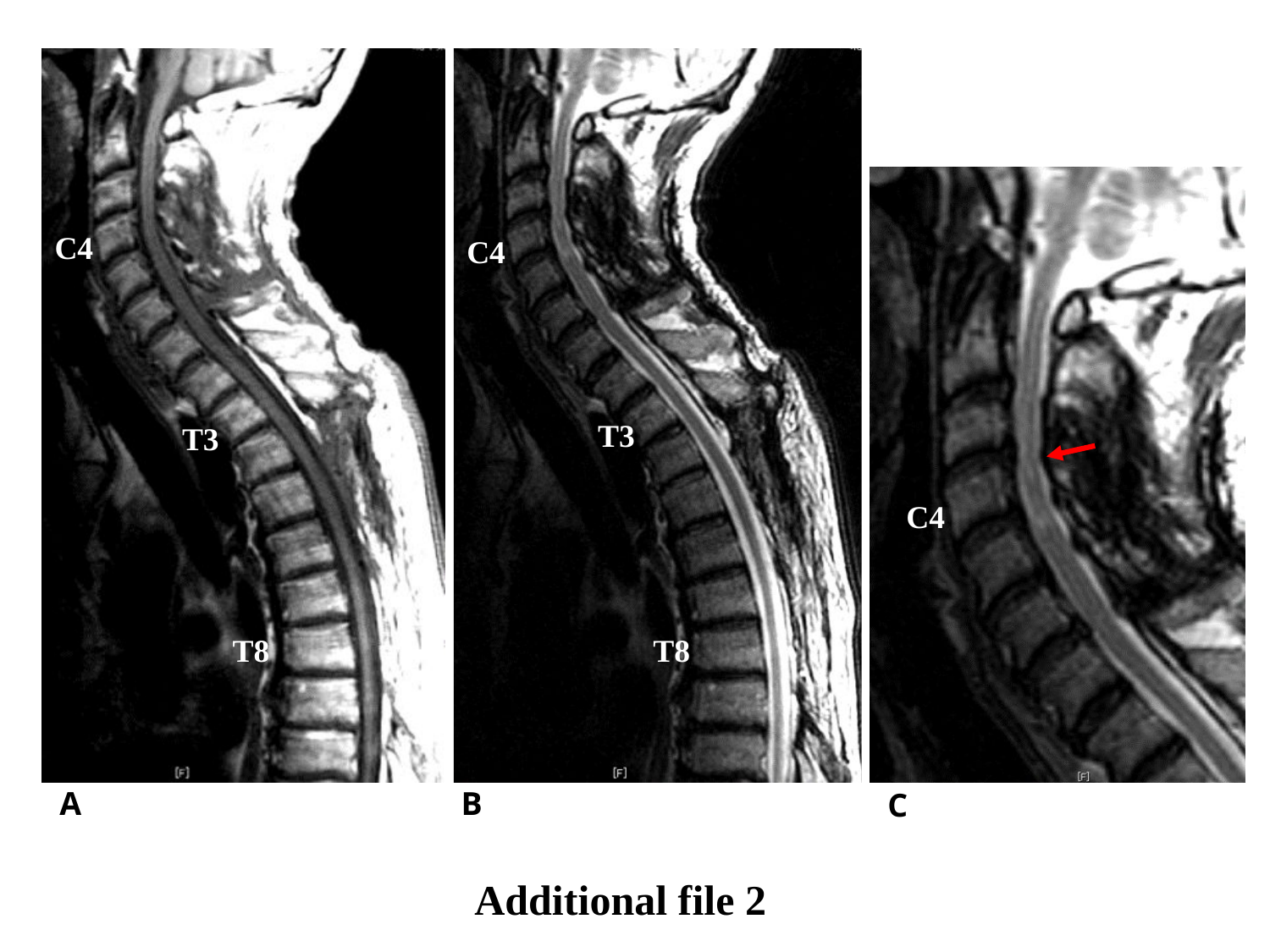

C4
C4
T3
T3
C4
T8
T8
A
B
C
Additional file 2

Supplement: Supplementary file 2 — Additional file 2: Magnetic resonance (MR) images of the cervical and thoracic spine 14 months after surgery. T1-weighted (A) and T2-weighted (B) midsagittal MR images show sufficient decompression of the spinal cord at the thoracic spine. Magnification of the T2-weighted image at the cervical spine area (C) shows slight posterior compression of the spinal cord at the C3–C4 level (red arrow). [file 13104_2022_5979_MOESM2_ESM.pptx]

## Slide 1
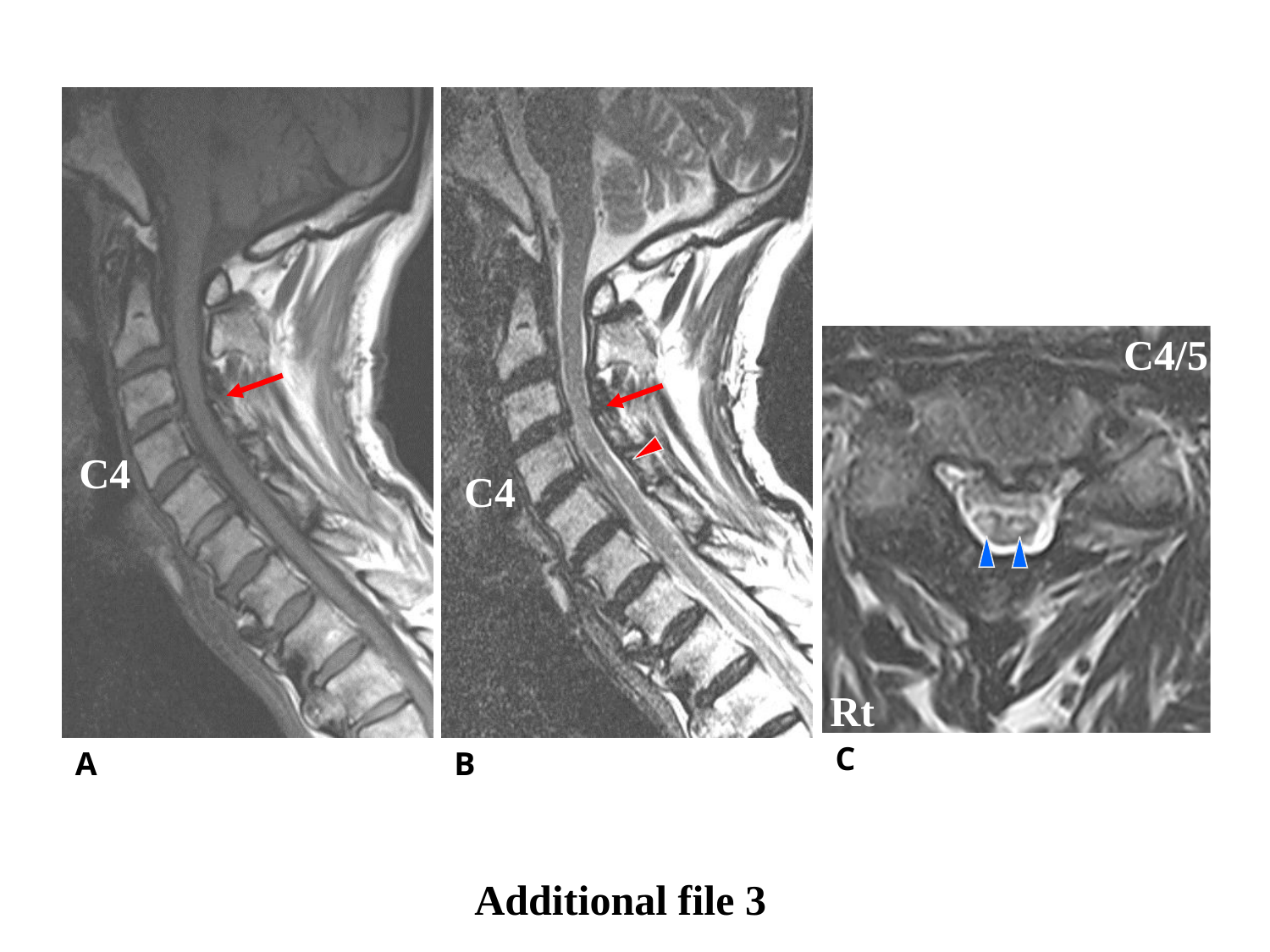

C4/5
C4
C4
C4
Rt
C
A
B
Additional file 3

Supplement: Supplementary file 3 — Additional file 3: MR images of the cervical spine 10 years and 6 months after surgery. T1-weighted (A) and T2-weighted (B) midsagittal MR images show slight posterior compression of the spinal cord at the C3–C4 level (red arrows). In the T2-weighted MR image (B), a high-intensity area was present inside the spinal cord at the C4–C5 level (red arrowhead), although the cord was thoroughly decompressed at this level. A T2-weighted MR axial image at the C4–C5 level (C) shows that the high-intensity area was predominant in the gray matter with a "snake-eyed" appearance (blue arrowheads). [file 13104_2022_5979_MOESM3_ESM.pptx]

## Slide 1
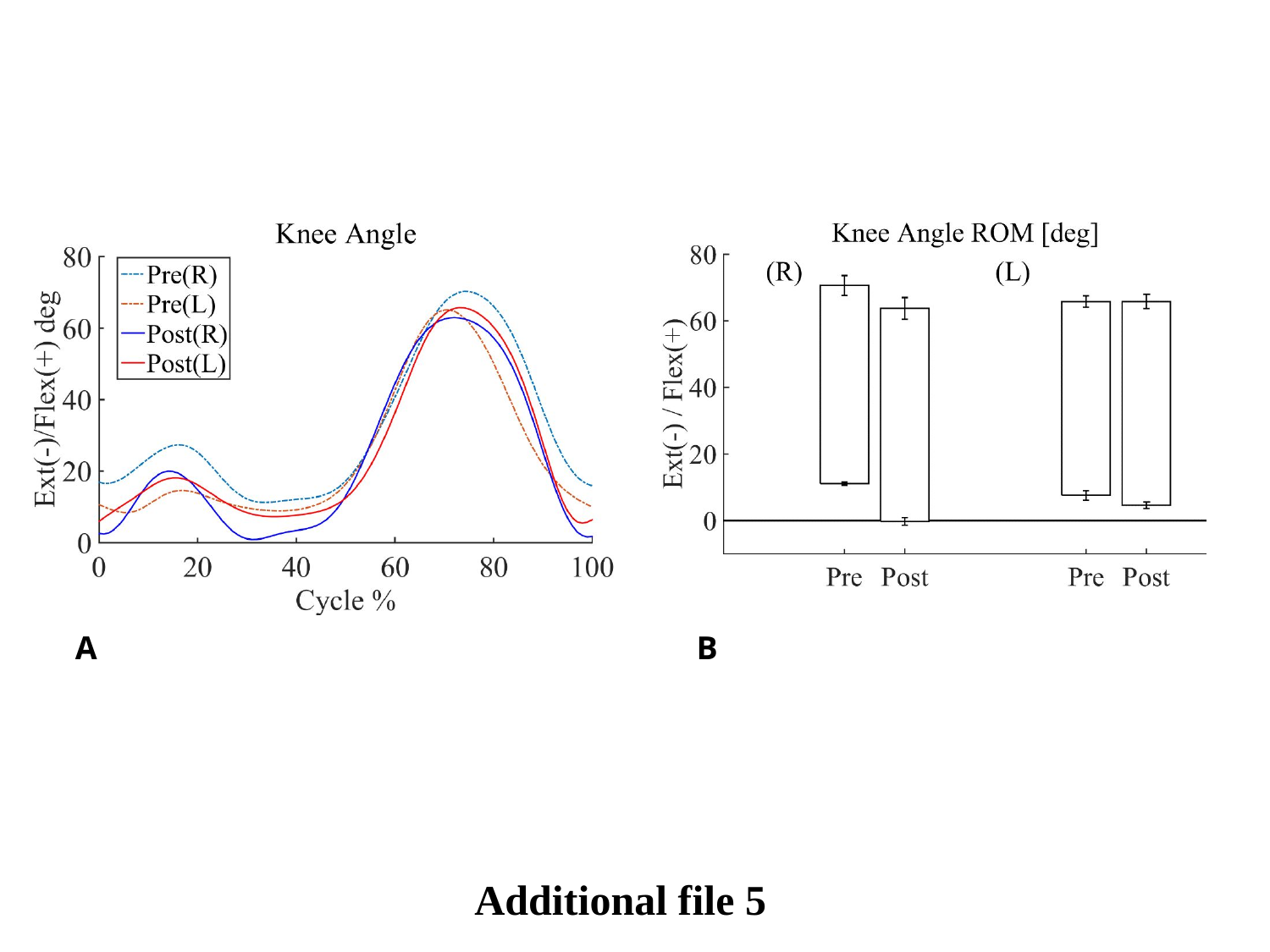

A
B
Additional file 5

## Slide 2
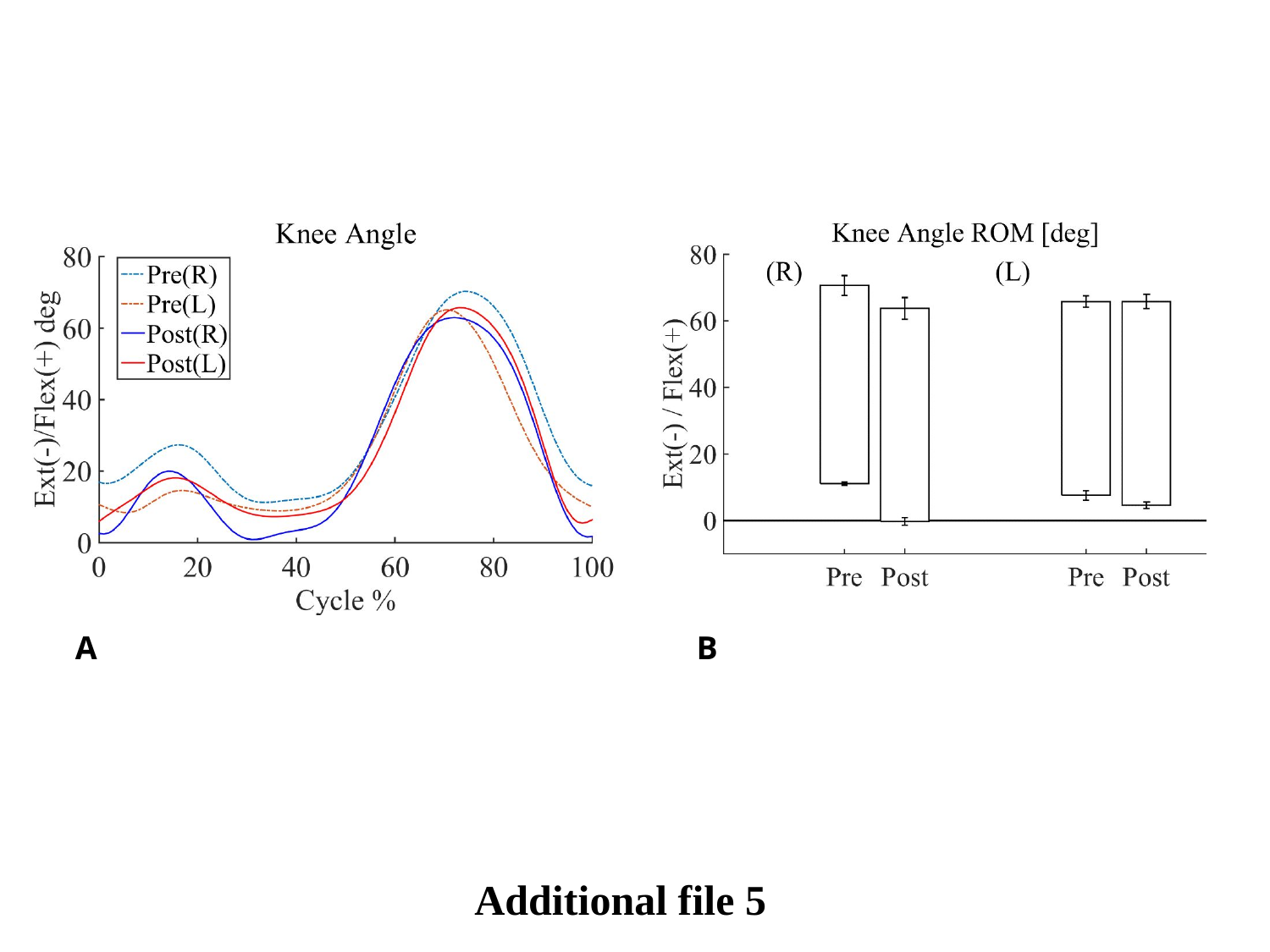

A
B
Additional file 5

Supplement: Supplementary file 5 — Additional file 5: Data of the kinematic motion analysis of the knee joint for HAL session 4. (A) Temporal profile of the angular position of the knee joint over the gait cycle (A) and range of motion of the knee over the gait cycle (B), measured without, immediately before, and after the HAL training. Error bars indicate standard error of the mean. Flex, flexion; Ext, extension; Pre, Pre-HAL training; Post, Post-HAL training; ROM, range of motion. [file 13104_2022_5979_MOESM5_ESM.pptx]

## Slide 1
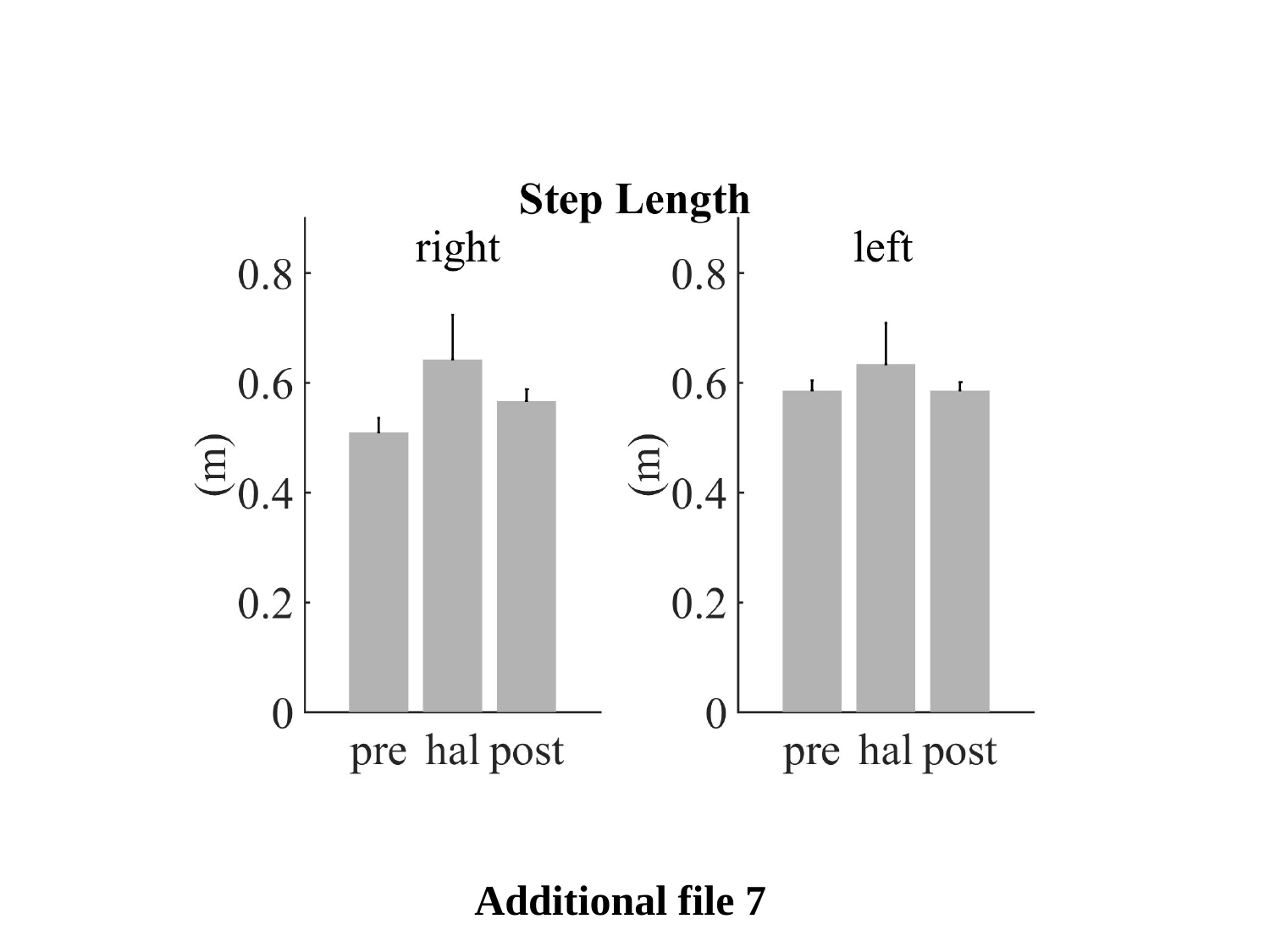

Additional file 7

Supplement: Supplementary file 7 — Additional file 7: Data of the kinematic motion analysis of step length for HAL session 4. Step length of the right and left legs immediately before and after HAL training. Pre, Pre-HAL training; Post, Post-HAL training. [file 13104_2022_5979_MOESM7_ESM.pptx]

## Slide 1
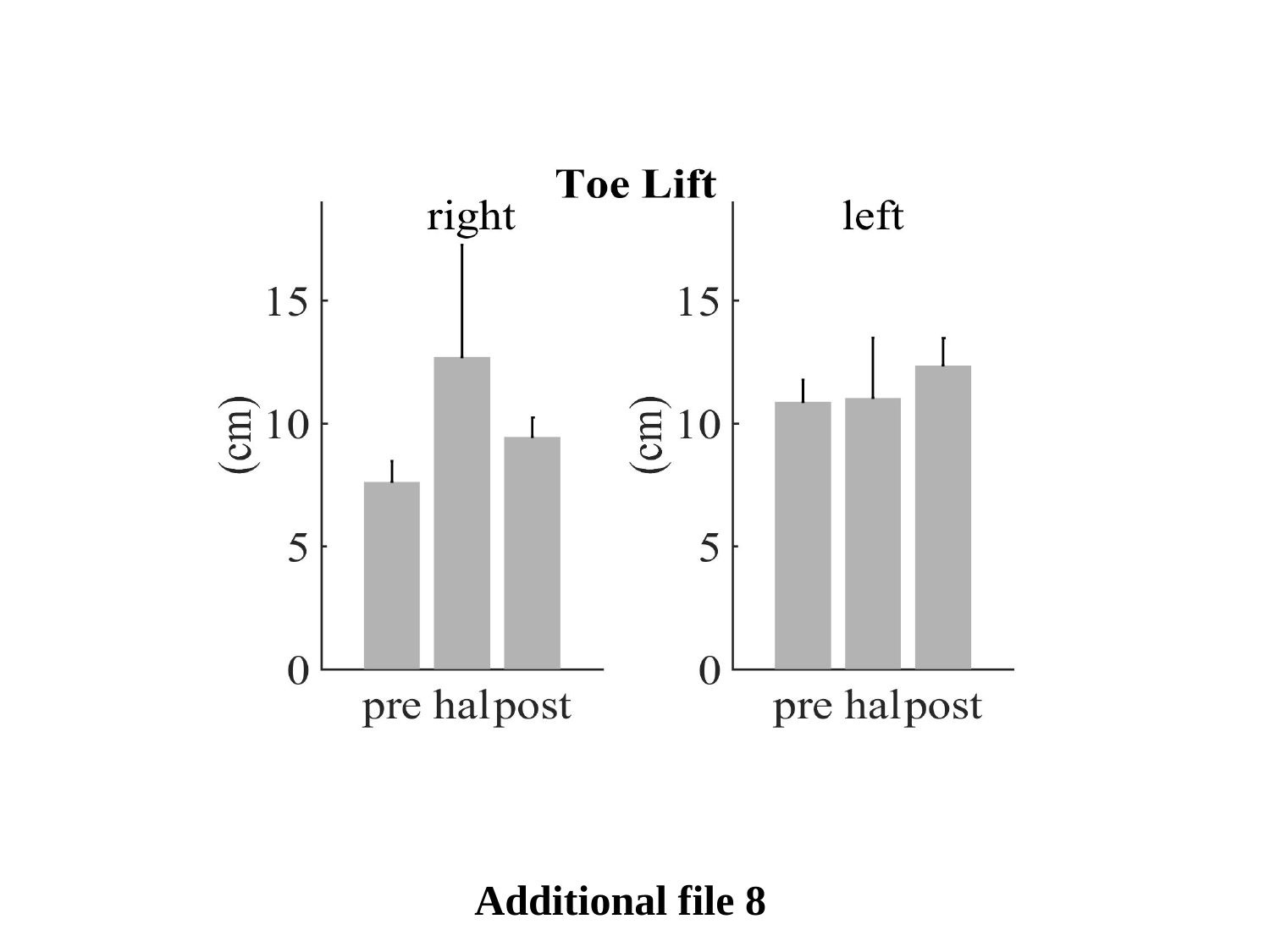

Additional file 8

Supplement: Supplementary file 8 — Additional file 8: Data of the kinematic motion analysis of toe clearance for HAL session 4. Toe lift of the right and left legs before, during, and after HAL gait training. The toe lift indicates toe clearance. Pre, Pre-HAL training; Post, Post-HAL training. [file 13104_2022_5979_MOESM8_ESM.pptx]

## Slide 1
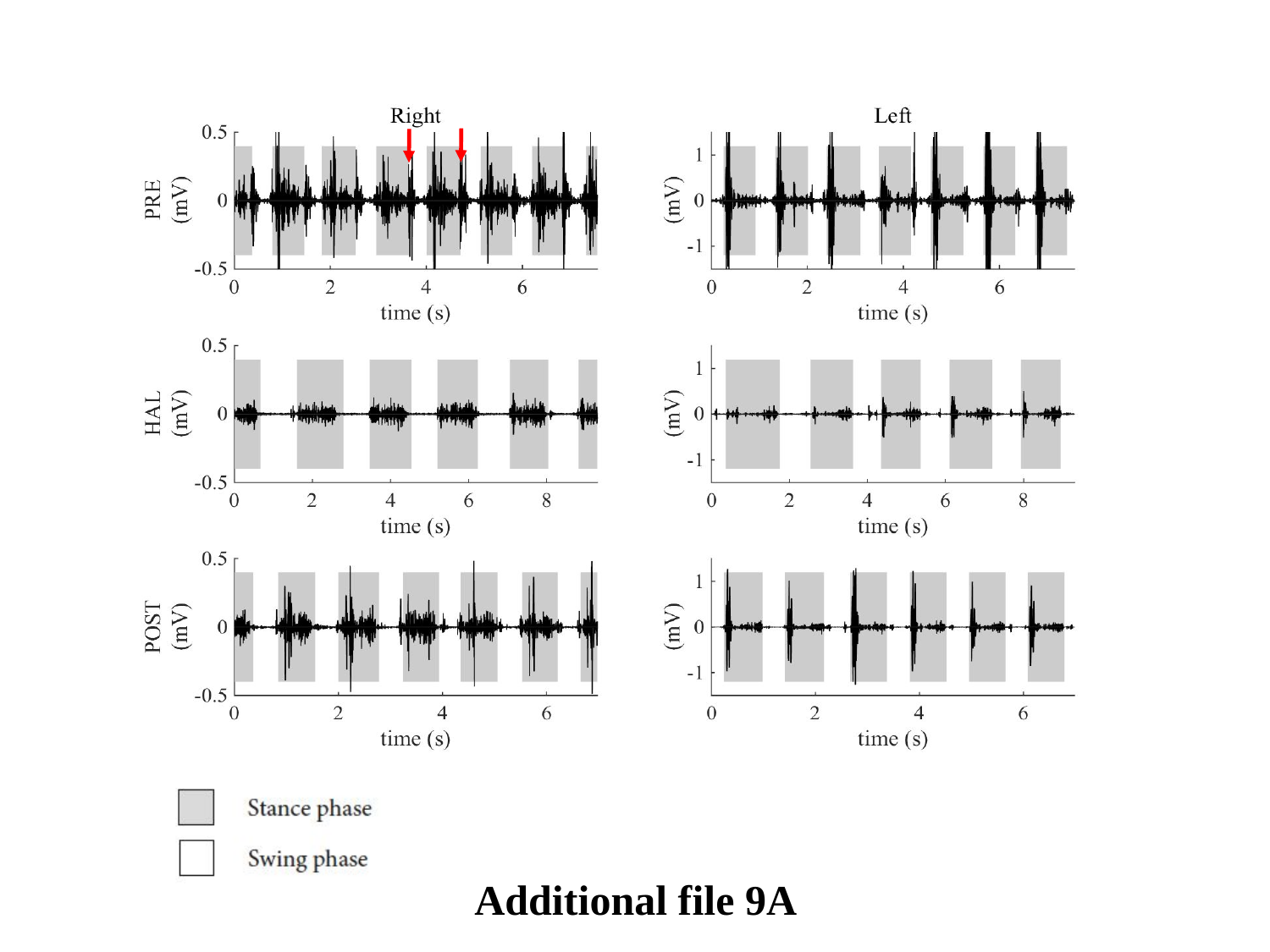

Additional file 9A

## Slide 2
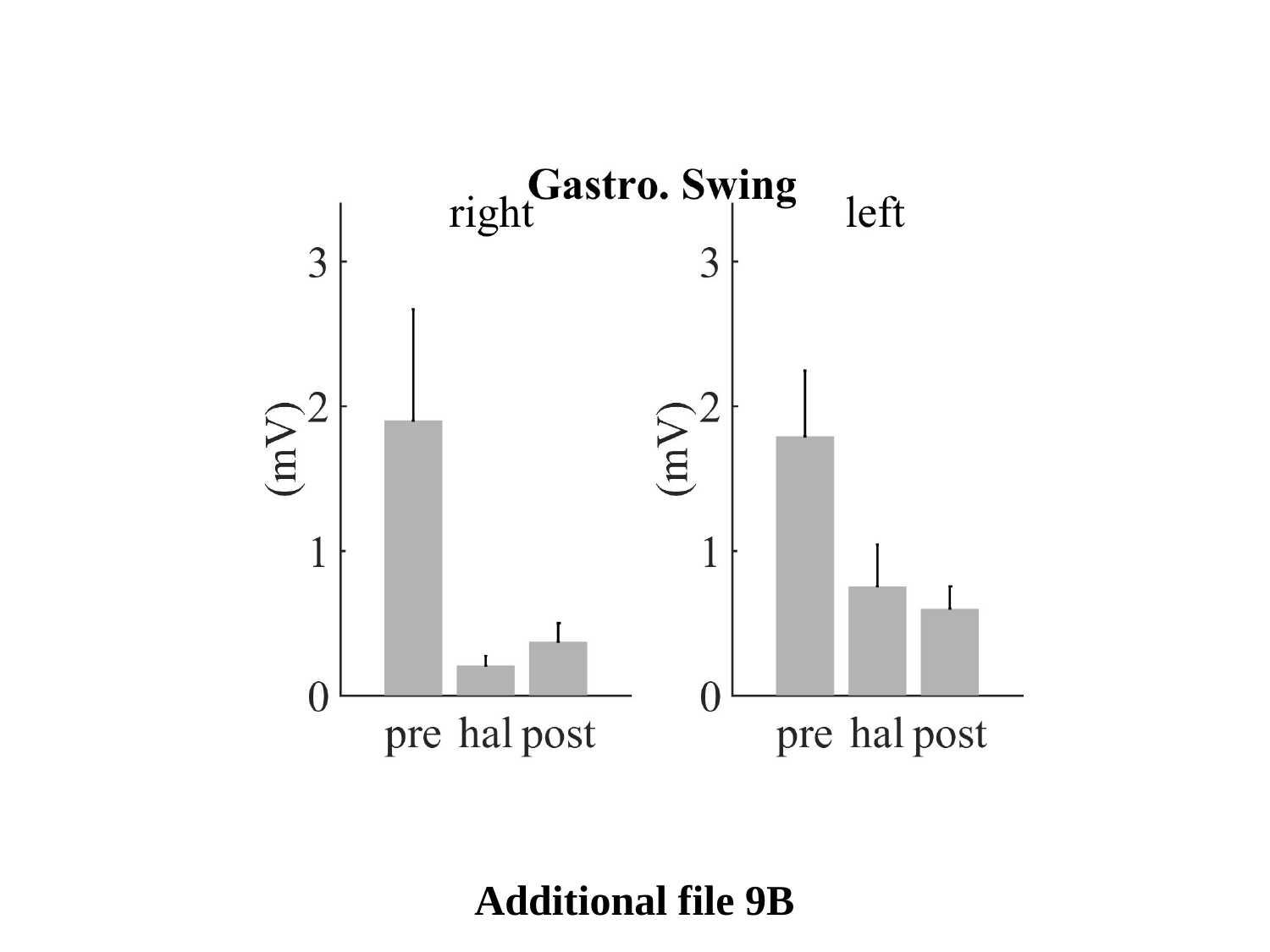

Additional file 9B

Supplement: Supplementary file 9 — Additional file 9: Kinematic motion analysis using the VICON motion capture system and surface electromyography (HAL session 4). (A) Surface electromyography of the gastrocnemius muscles of the right and left legs during the stance and swing phases. (B) Gastrocnemius stance ratio of the right and left legs before, during, and after the HAL gait training. The gastrocnemius stance ratio indicates the muscle activation ratio of the gastrocnemius (swing phase to the total step cycle). Pre, Pre-HAL training; Post, Post-HAL training. [file 13104_2022_5979_MOESM9_ESM.pptx]
