# Supplementary material for: Immediate effects of hybrid assistive limb gait training on lower limb function in a chronic myelopathy patient with postoperative late neurological deterioration
Source: BMC Res Notes. 2022 Mar 4;15:89. doi: 10.1186/s13104-022-05979-4 (PMC8896224; doi:10.1186/s13104-022-05979-4)
Supplement: Supplementary file 4 — Additional file 4: Data of the kinematic motion analysis of the hip joint for HAL session 4. (A) Temporal profile of the angular position of the hip joint over the gait cycle (A) and range of motion of the hip over the gait cycle (B), measured without, immediately before, and after the HAL training. Error bars indicate standard error of the mean. Flex, flexion; Ext, extension; Pre, Pre-HAL training; Post, Post-HAL training; ROM, range of motion. [file 13104_2022_5979_MOESM4_ESM.pptx]

## Slide 1
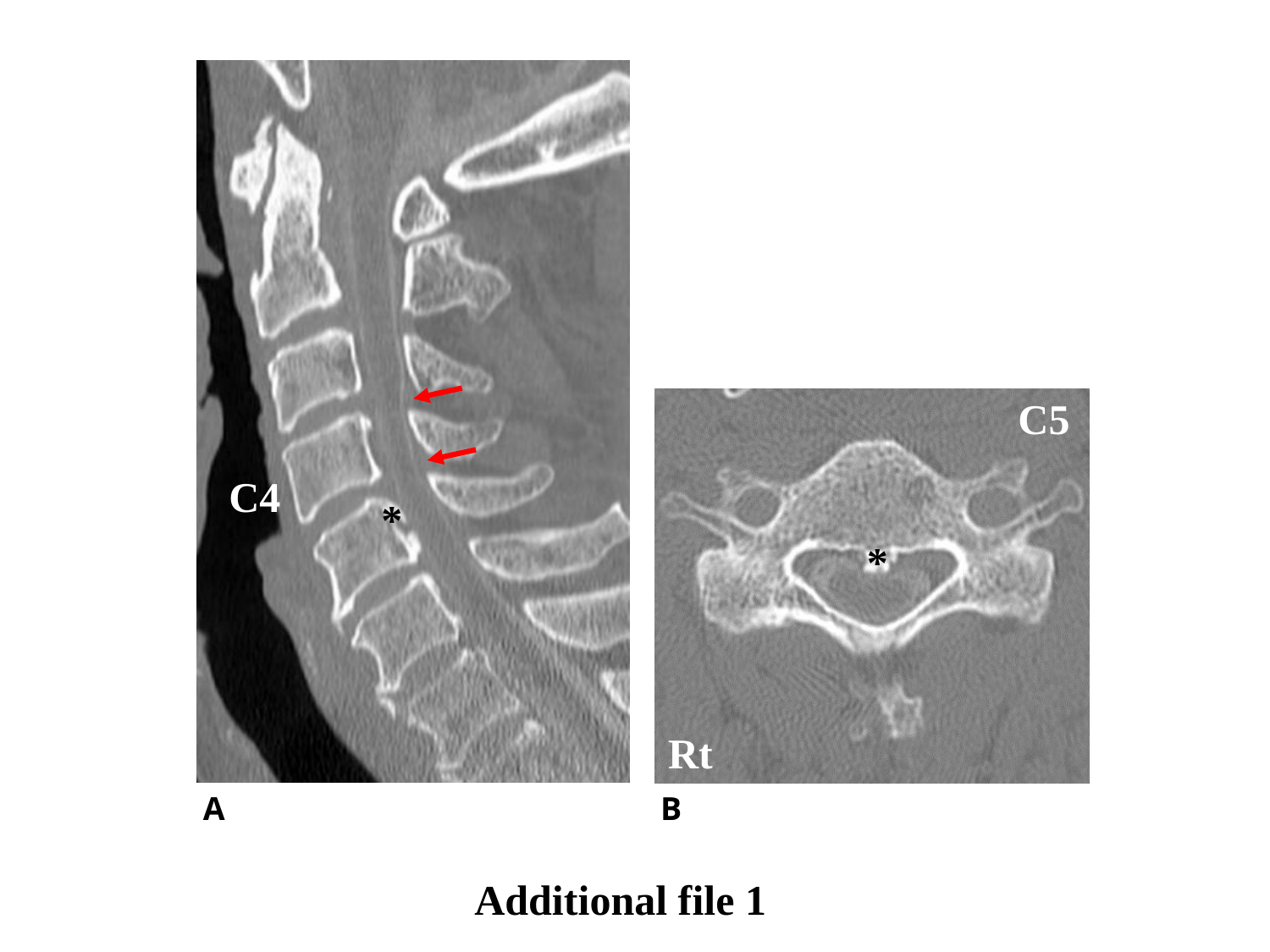

C5
C4
*
*
Rt
A
B
Additional file 1

## Slide 2
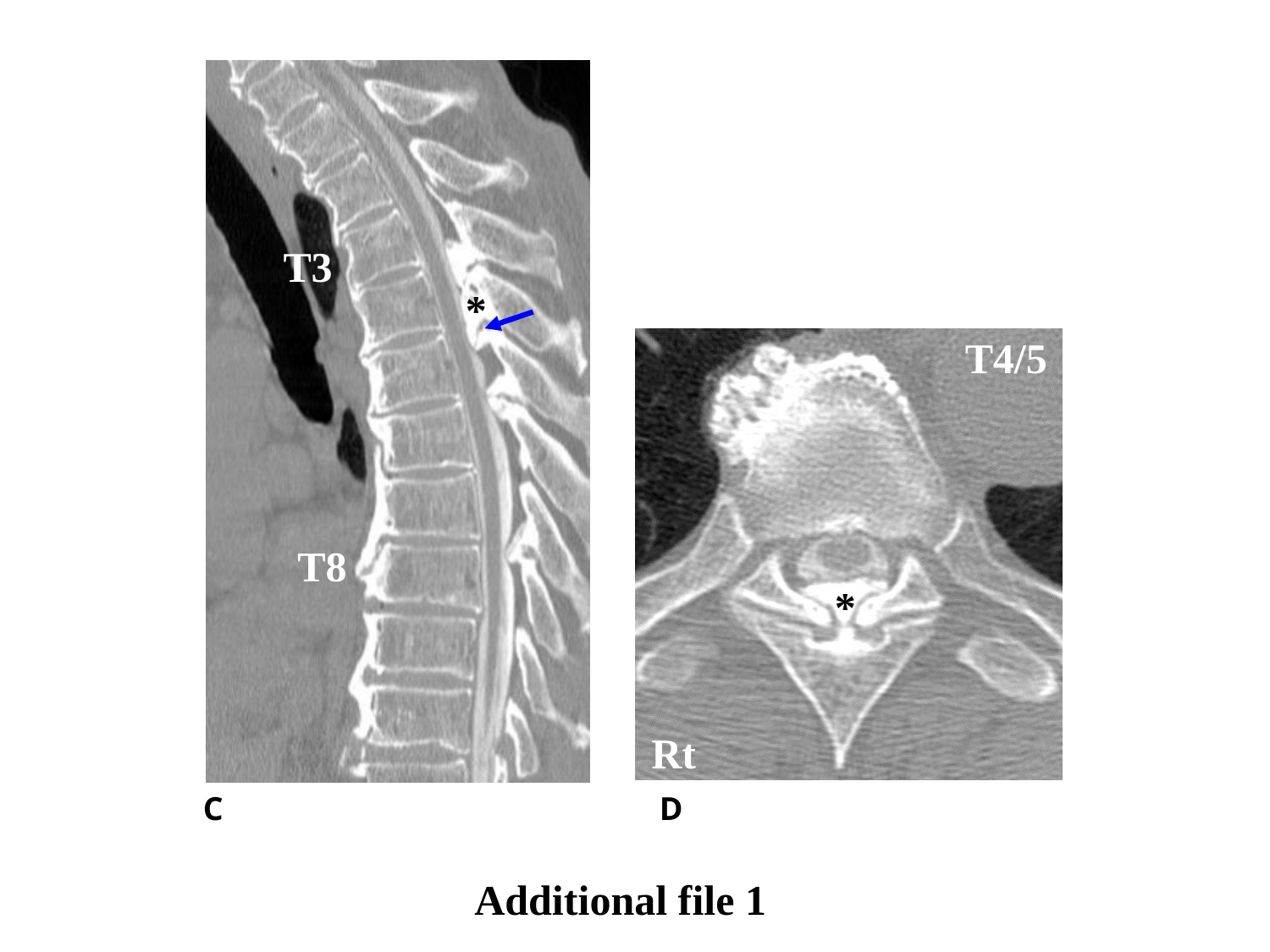

T3
*
T4/5
T8
*
Rt
C
D
Additional file 1

## Slide 3
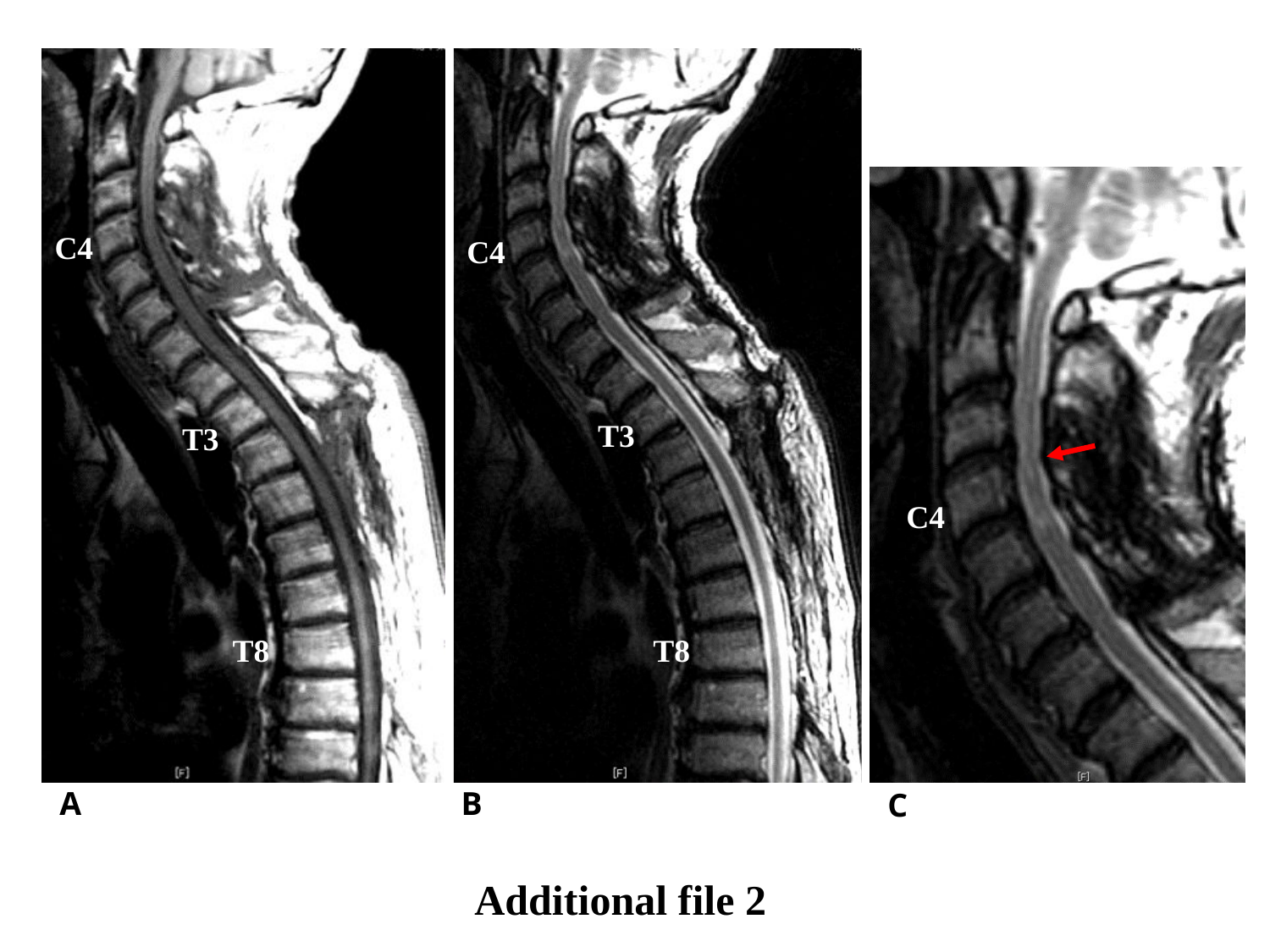

C4
C4
T3
T3
C4
T8
T8
A
B
C
Additional file 2

## Slide 4
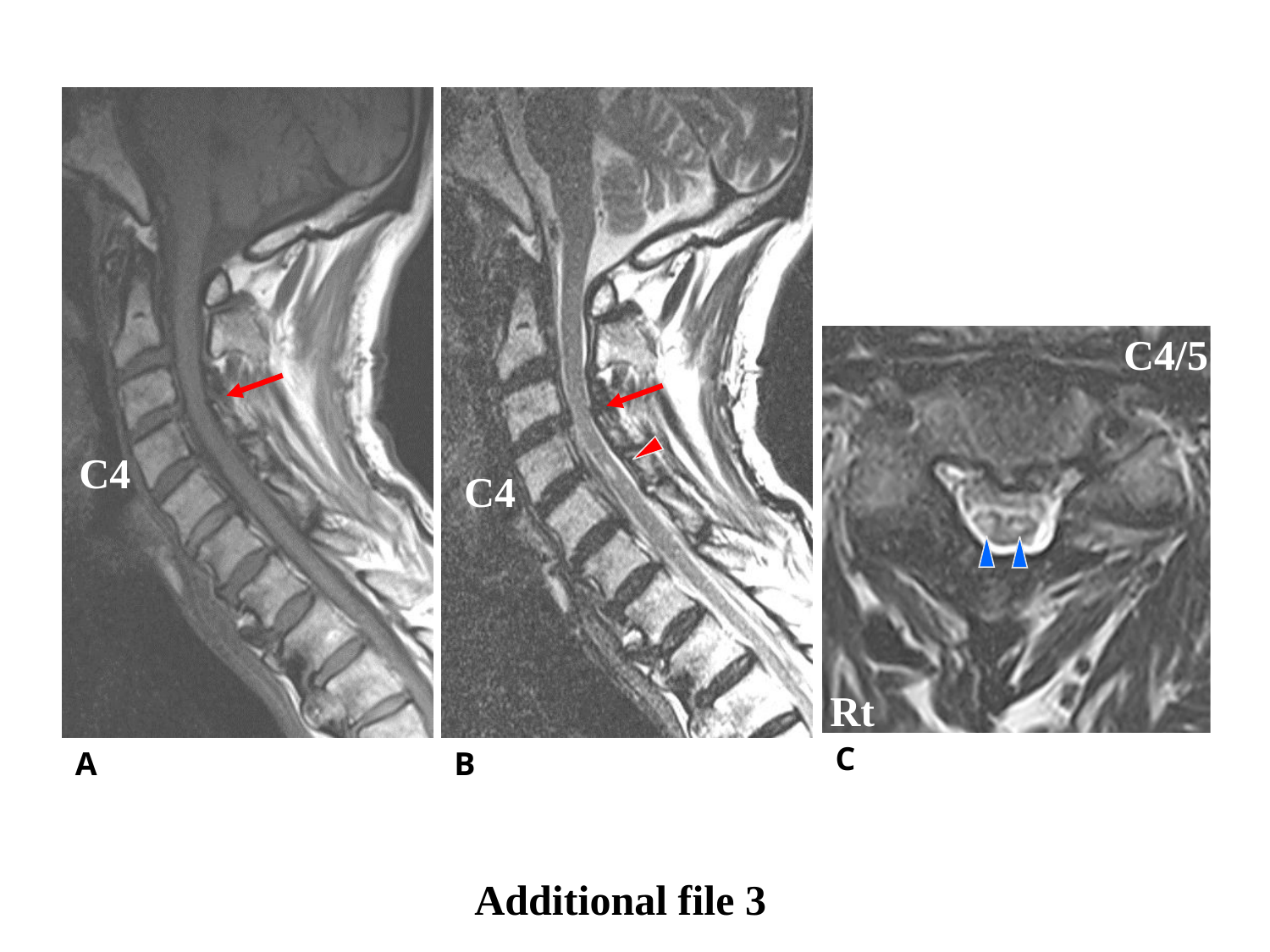

C4/5
C4
C4
C4
Rt
C
A
B
Additional file 3

## Slide 5
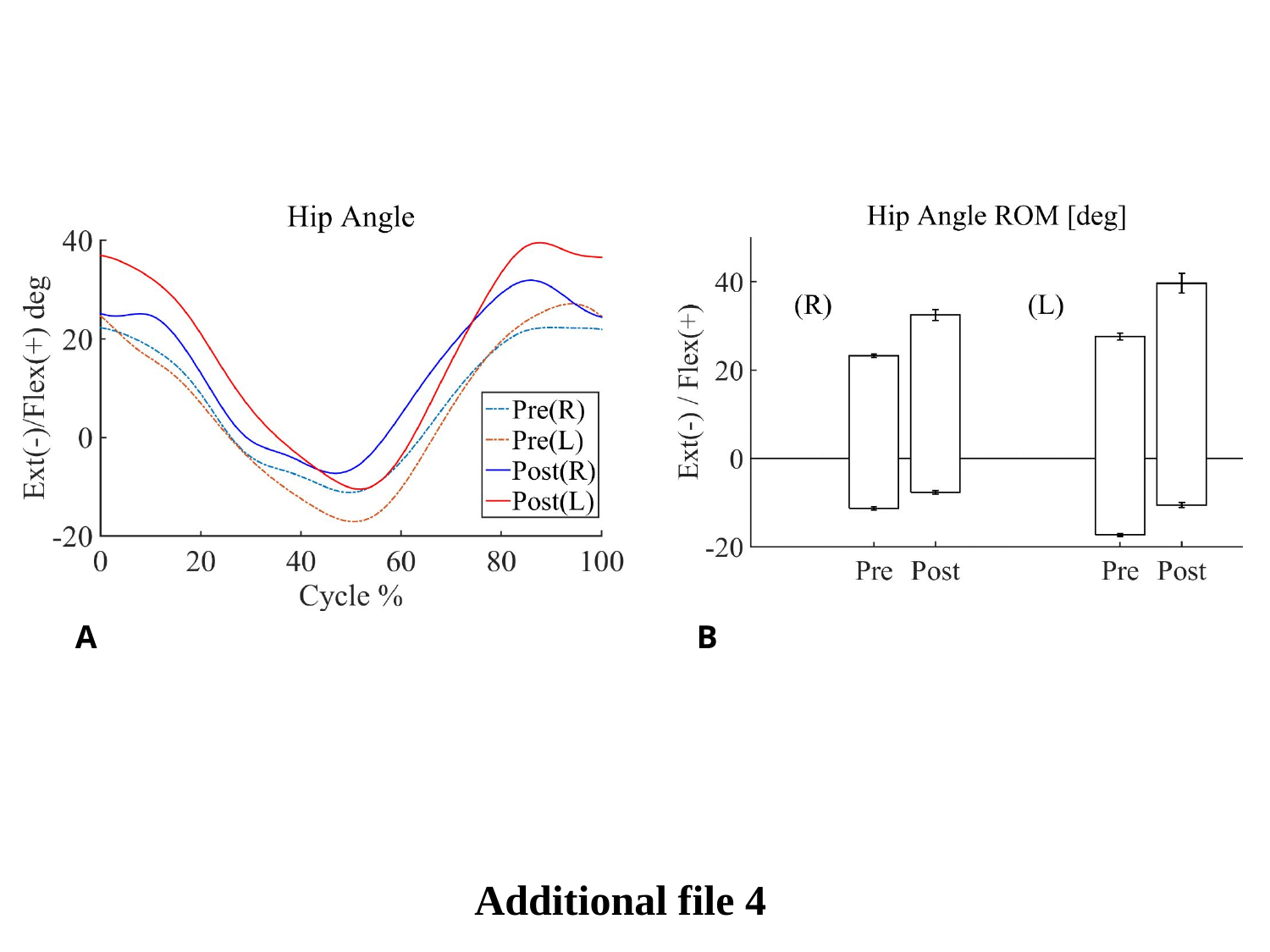

A
B
Additional file 4

## Slide 6
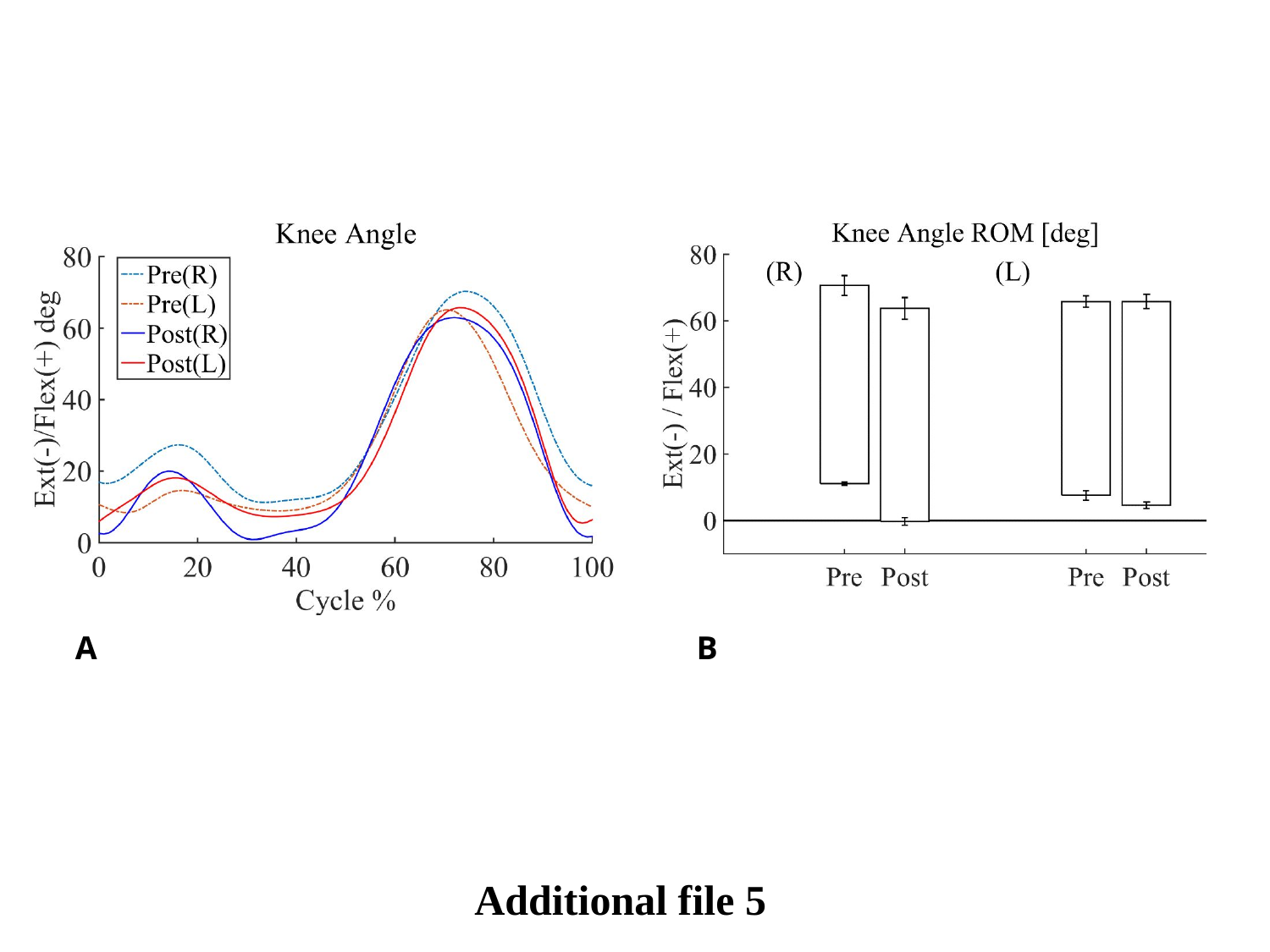

A
B
Additional file 5

## Slide 7
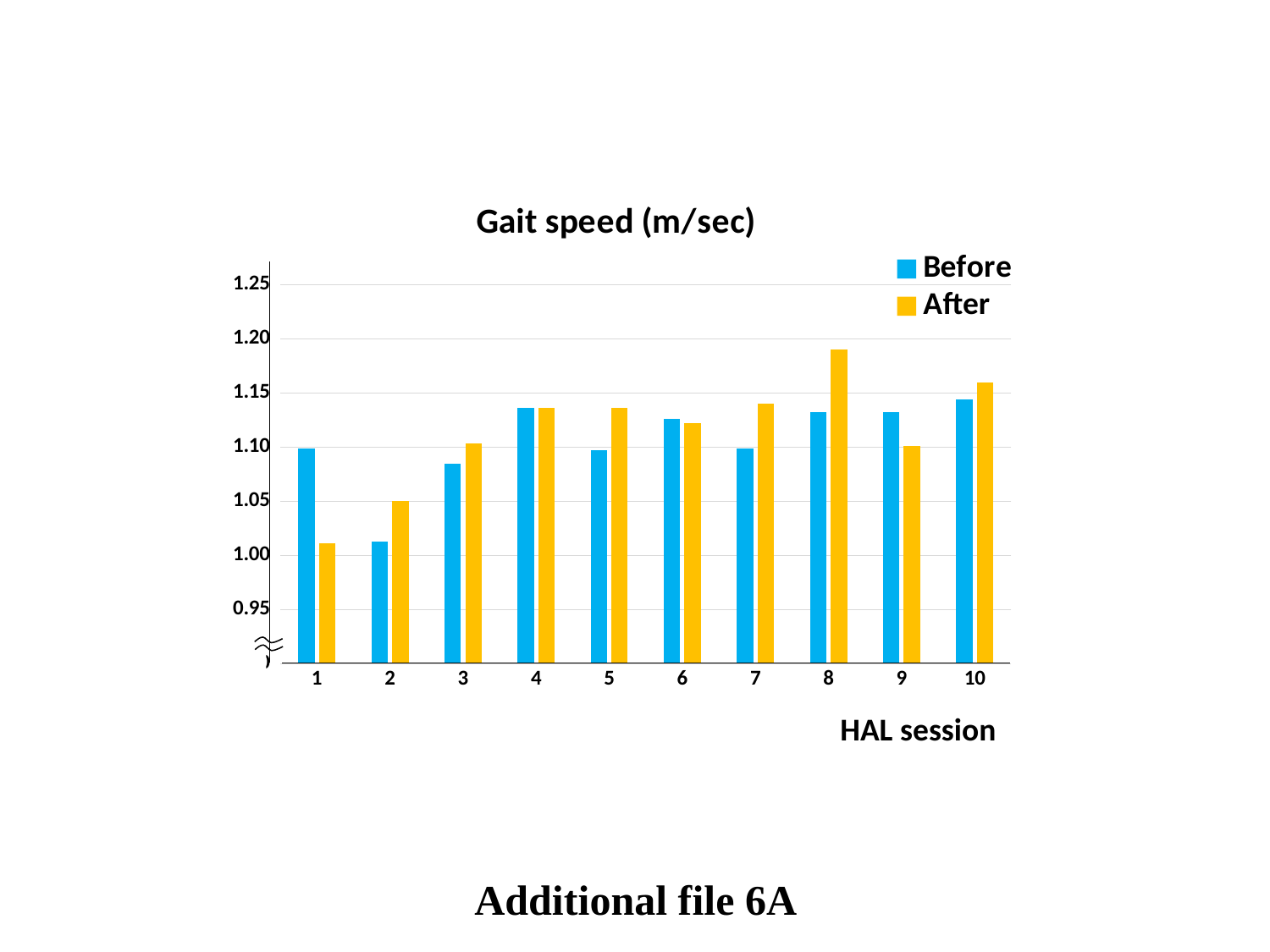

### Chart: Gait speed (m/sec)
| Category | | |
|---|---|---|
HAL session
Additional file 6A

## Slide 8
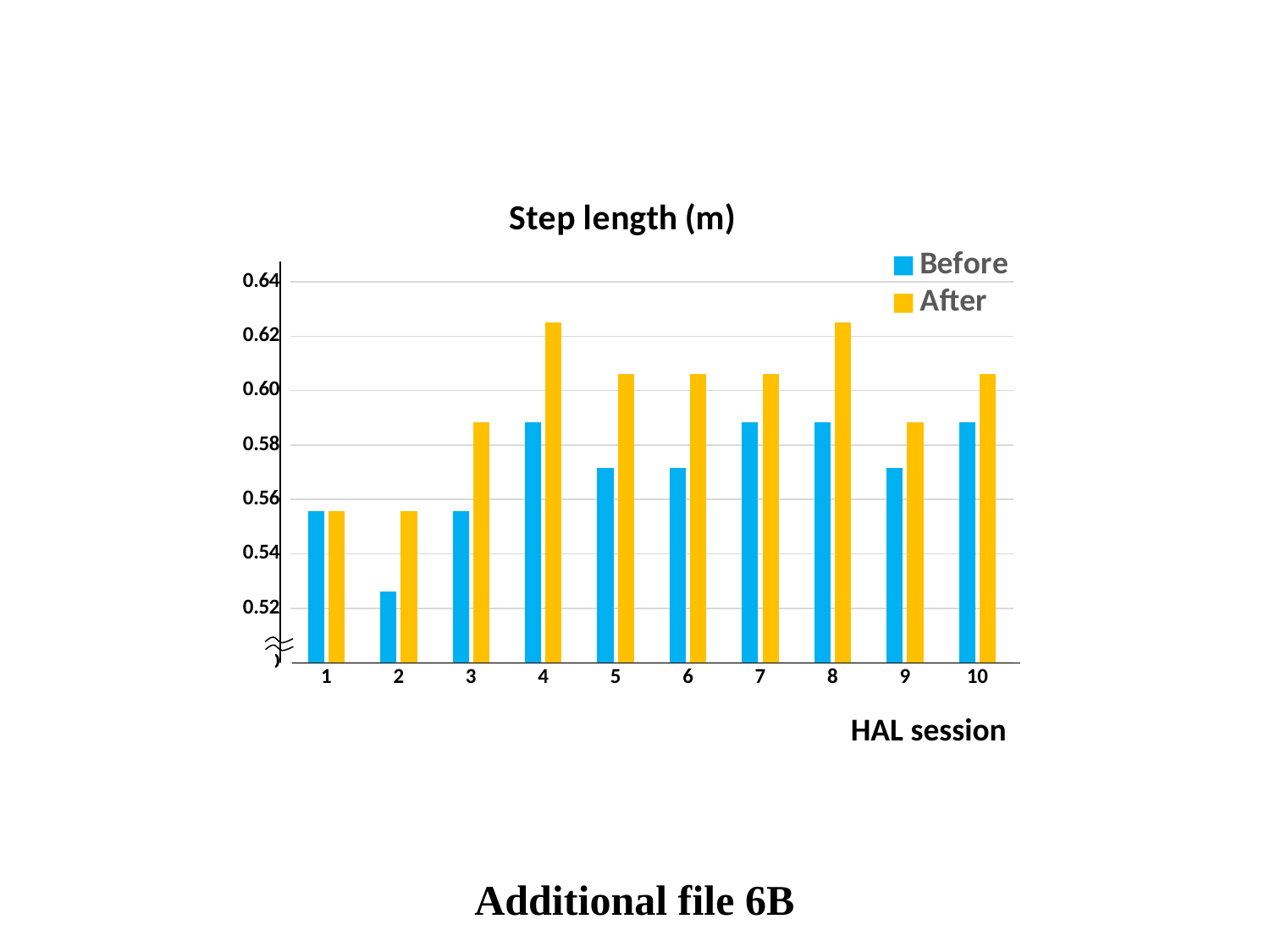

### Chart: Step length (m)
| Category | | |
|---|---|---|
HAL session
Additional file 6B

## Slide 9
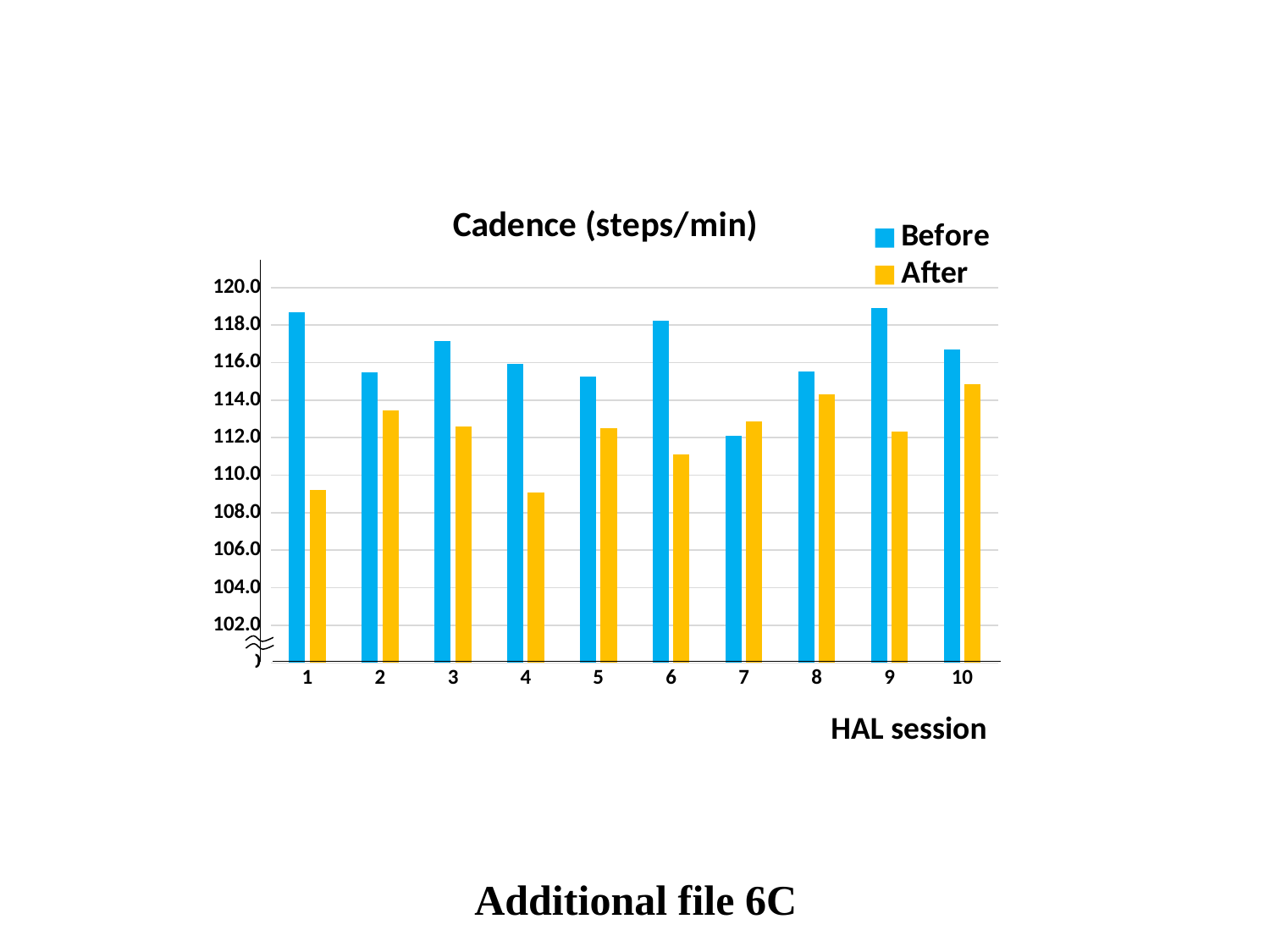

### Chart: Cadence (steps/min)
| Category | | |
|---|---|---|
HAL session
Additional file 6C

## Slide 10
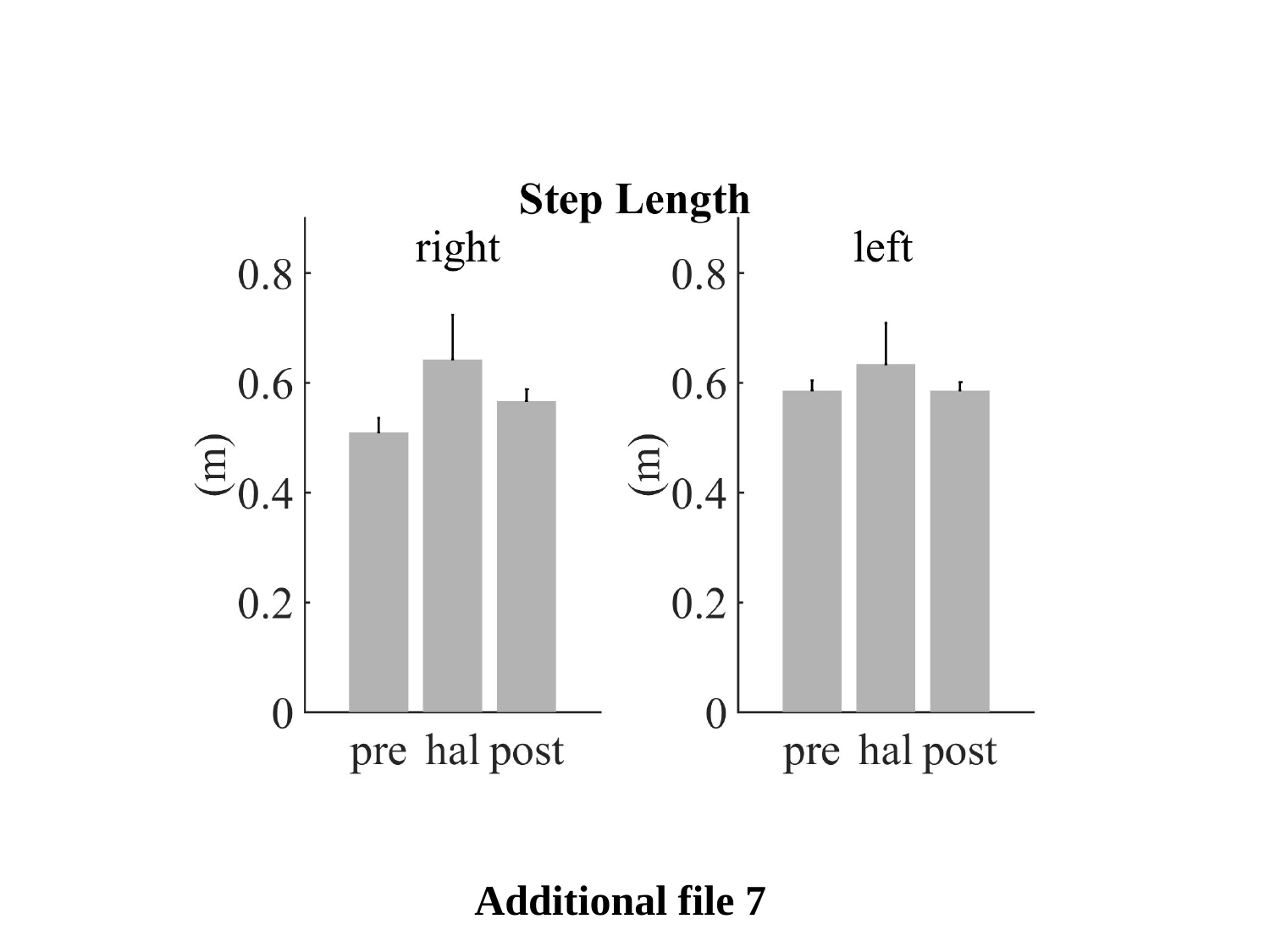

Additional file 7

## Slide 11
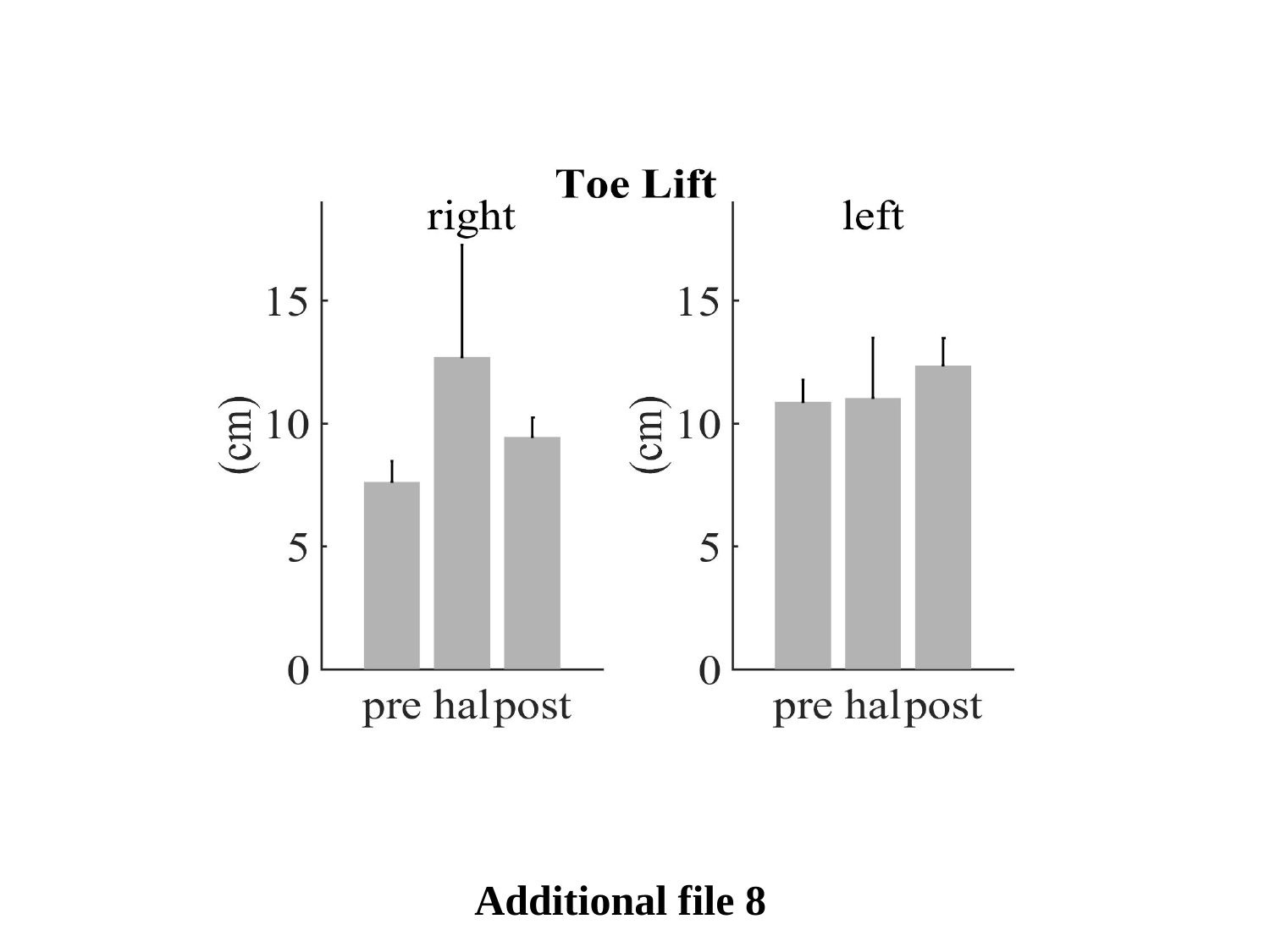

Additional file 8

## Slide 12
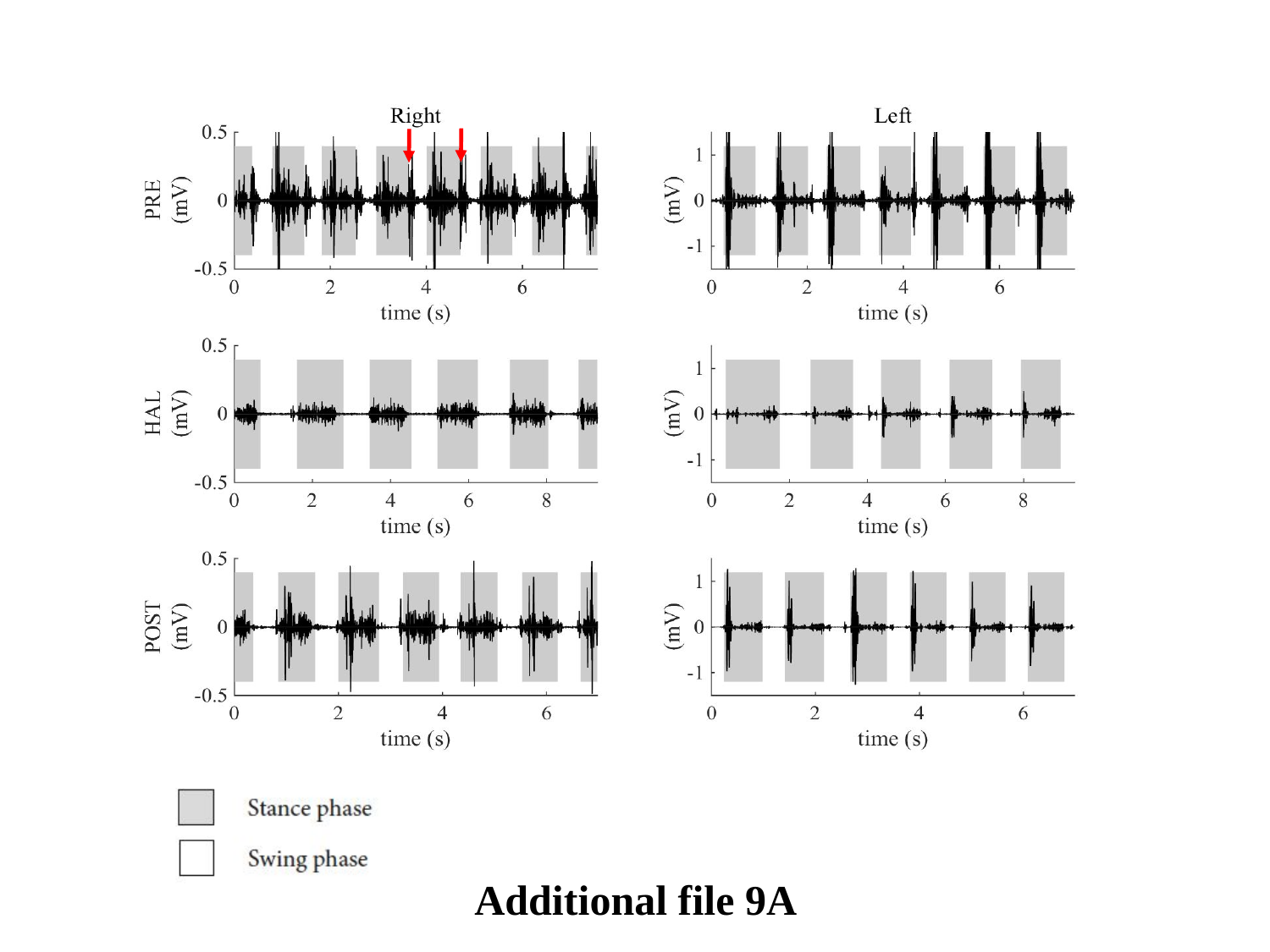

Additional file 9A

## Slide 13
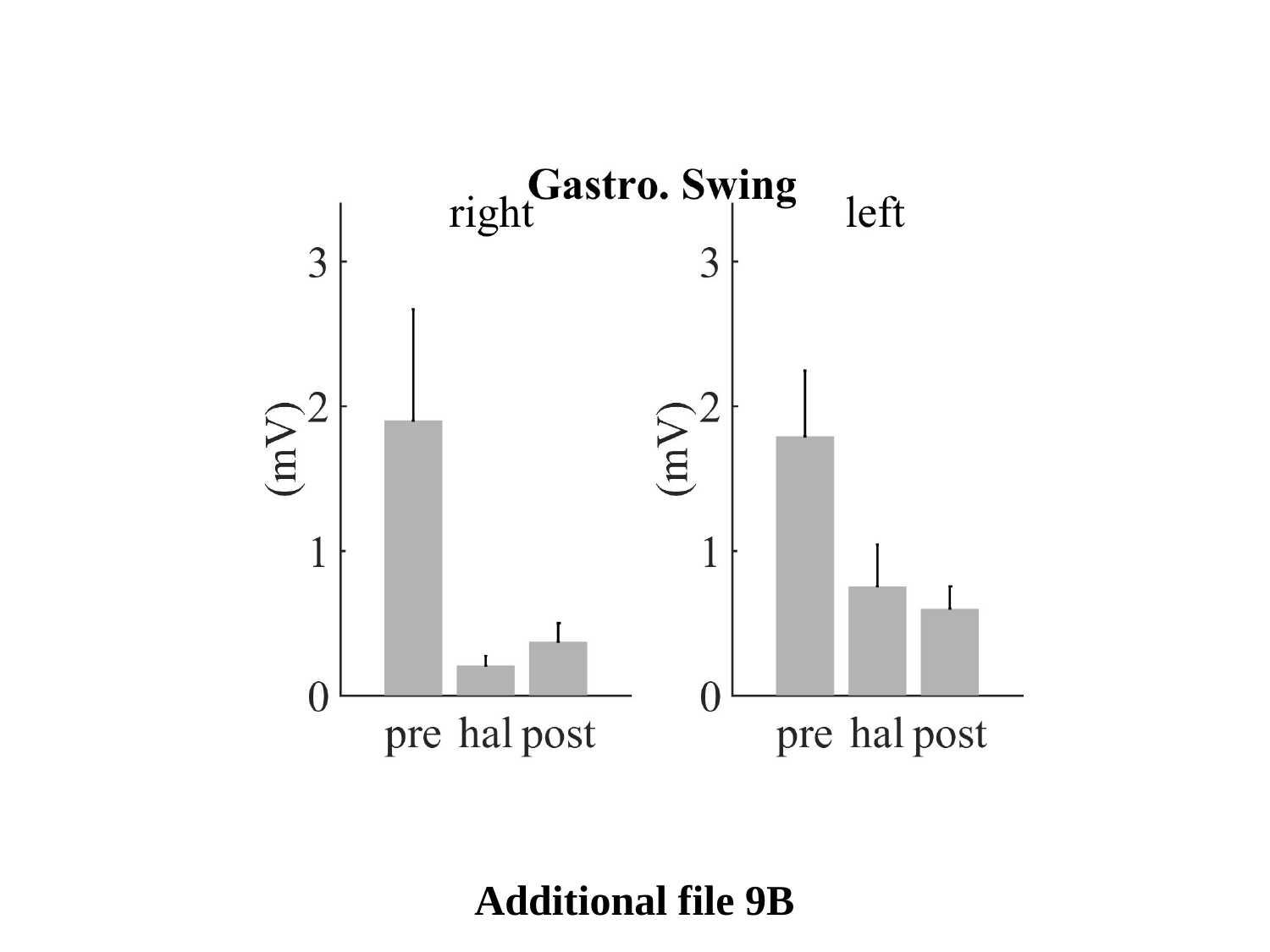

Additional file 9B
