# Supplementary material for: Immediate effects of hybrid assistive limb gait training on lower limb function in a chronic myelopathy patient with postoperative late neurological deterioration
Source: BMC Res Notes. 2022 Mar 4;15:89. doi: 10.1186/s13104-022-05979-4 (PMC8896224; doi:10.1186/s13104-022-05979-4)
Supplement: Supplementary file 6 — Additional file 6: The 10-m walk test data immediately before and after HAL gait training, sessions 1–10. Gait speed (A), step length (B), and cadence (C). [file 13104_2022_5979_MOESM6_ESM.pptx]

## Slide 1
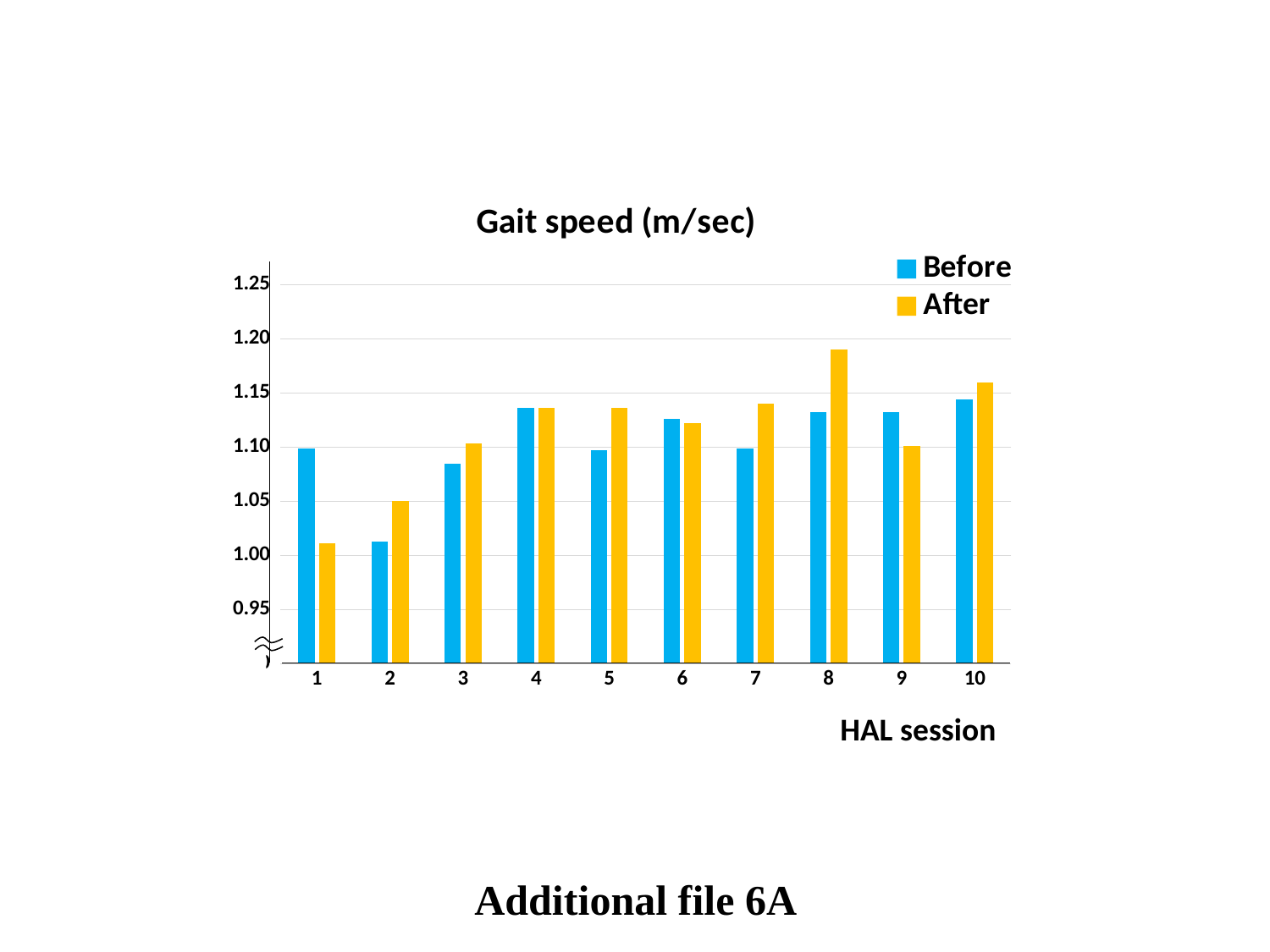

### Chart: Gait speed (m/sec)
| Category | | |
|---|---|---|
HAL session
Additional file 6A

## Slide 2
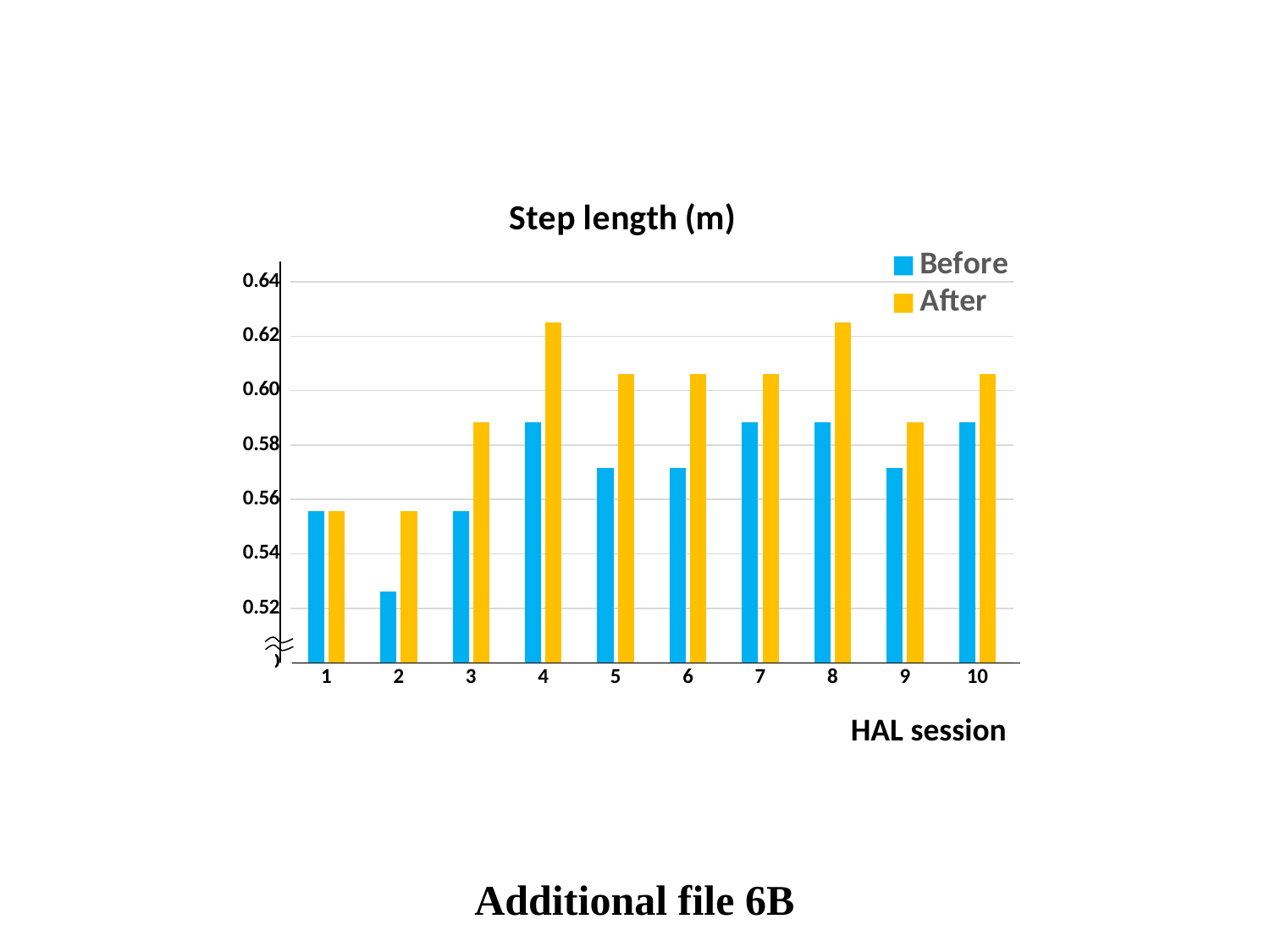

### Chart: Step length (m)
| Category | | |
|---|---|---|
HAL session
Additional file 6B

## Slide 3
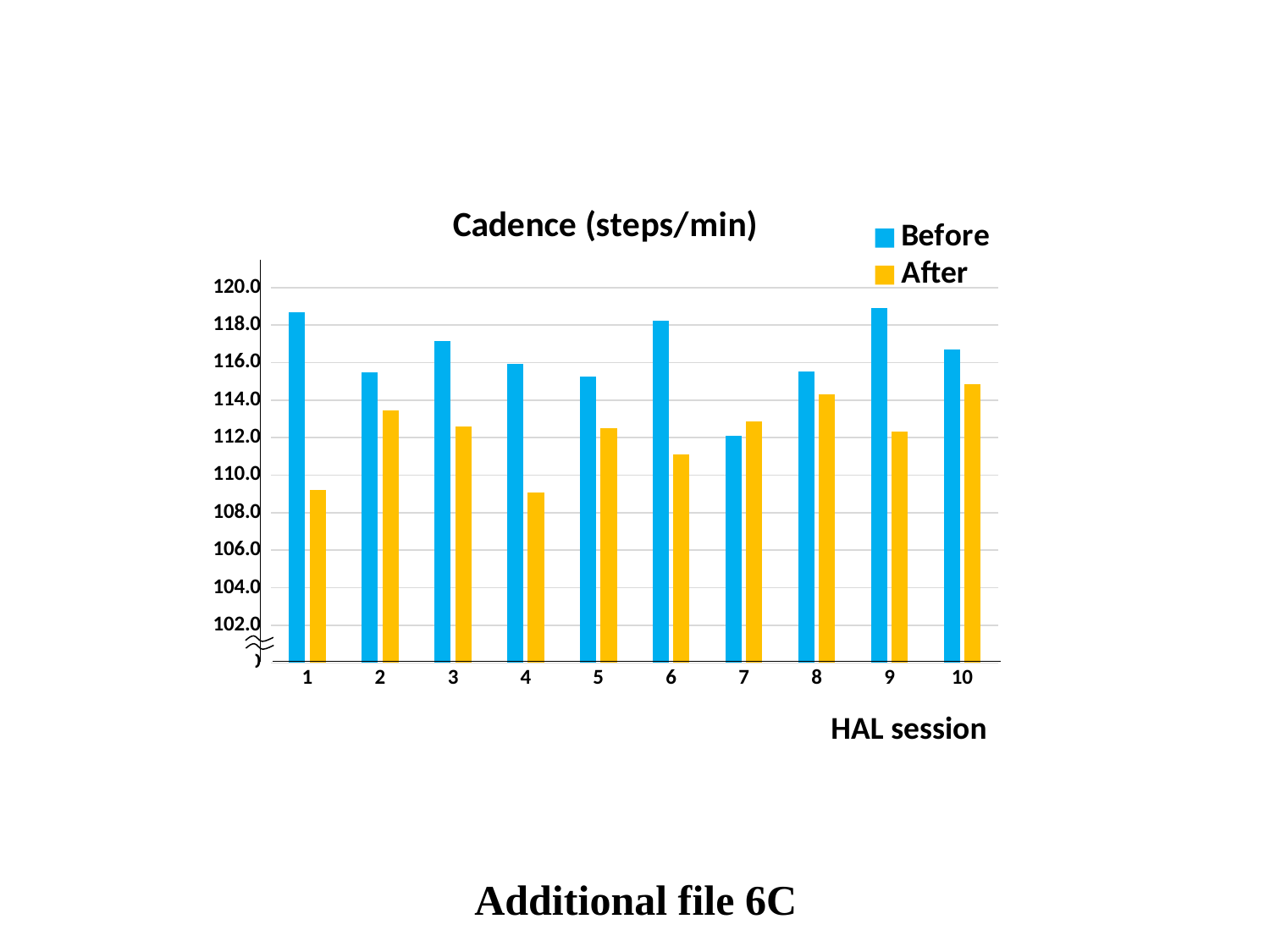

### Chart: Cadence (steps/min)
| Category | | |
|---|---|---|
HAL session
Additional file 6C
